# Crosslinked-AuNPs@CD-MOF Incorporated into PLA-Zein Composite Film with Humidity-Responsive Antimicrobial Release for *Agaricus bisporus* Preservation

**DOI:** 10.3390/foods15071164

**Published:** 2026-03-30

**Authors:** Tahirou Sogore, Meimei Guo, Jin Huang, Xinyu Liao, Tian Ding, Mofei Shen

**Affiliations:** 1 College of Biosystems Engineering and Food Science, Zhejiang University, Hangzhou 310058, China; 12013074@zju.edu.cn (T.S.);; 2 Future Food Laboratory, Innovation Center of Yangtze River Delta, Zhejiang University, Jiaxing 314100, China; 3 Department of Food Science and Engineering, Zhejiang University of Technology, Hangzhou 310014, China

**Keywords:** AuNPs, cyclodextrin metal–organic framework, crosslinking, zein/polylactic acid, food quality control, antimicrobial

## Abstract

Foodborne pathogens cause hundreds of millions of illnesses annually, underscoring the urgent need for advanced antimicrobial food packaging materials. The objective of this study was to develop a crosslinked cyclodextrin metal–organic framework, loaded with gold nanoparticles (CL-AuNPs@CD-MOF) and integrated into a PLA-Zein composite film with humidity-responsive antimicrobial release, as a sustainable and high-performance packaging solution to address the critical limitations of conventional materials in controlling microbial contamination during food storage. Therefore, gold nanoparticles (AuNPs) were synthesized via a green approach using CD-MOFs as stabilizers and p-coumaric acid as a natural reducing agent, then crosslinked with diphenyl carbonate (DPC) to produce CL-AuNPs@CD-MOF. Crosslinking conditions were optimized to a CD-MOF:DPC ratio of 1:1, 1080 min reaction time, and 80 °C, preserving the cubic morphology and crystalline structure while transforming burst release into sustained antimicrobial activity against *E. coli* and *S. aureus* over 7 days. Then, the incorporation of CL-AuNPs@CD-MOF into PLA-Zein films yielded a composite packaging material with favorable mechanical and barrier properties, including a water vapor transmission rate of 539.44 g/m^2^·24 h and an oxygen permeability of 235.90 cm^3^/m^2^·24 h·0.1 MPa. Progressive elimination of *E. coli*, *S. aureus*, and *L. monocytogenes* over 7 days was confirmed, with antimicrobial efficacy originating exclusively from the CL-AuNPs@CD-MOF component. Application on *Agaricus bisporus* over 12 days of refrigerated storage demonstrated superior preservation performance: mushrooms inoculated with *L. monocytogenes* and packaged with CL-AuNPs@CD-MOF/PLA-Zein exhibited a weight loss of only 6.20 ± 2.06%, compared to 17.74 ± 3.15% for PLA-Zein and 41.50 ± 3.01% for PE controls. Color stability was equally improved, with lightness values of 71.46 ± 1.47 retained under CL-AuNPs@CD-MOF/PLA-Zein packaging, versus 58.37 ± 0.86 for PLA-Zein and 23.34 ± 2.34 for PE. Mushrooms inoculated with *E. coli* and *S. aureus* followed consistent trends. These results establish CL-AuNPs@CD-MOF/PLA-Zein as a promising multifunctional antimicrobial packaging platform for sustainable food preservation.

## 1. Introduction

Food spoilage caused by microorganisms represents one of the most critical challenges in the global food supply chain, resulting in substantial economic losses, nutritional waste, and serious public health risks worldwide. It is estimated that approximately one-third of all food produced globally is lost or wasted each year, with microbial spoilage accounting for a significant proportion of these losses, particularly in fresh and minimally processed products [1]. Among the most vulnerable commodities, fresh mushrooms such as *Agaricus bisporus* are highly perishable due to their elevated moisture content, active enzymatic metabolism, and thin, unprotected cuticle, making them extremely susceptible to rapid microbial colonization post-harvest [2]. Common spoilage microorganisms, including bacteria such as *Pseudomonas* spp., *Bacillus* spp., and *Leuconostoc* spp., as well as various molds and yeasts, proliferate rapidly on mushroom surfaces under ambient storage conditions, leading to browning, texture deterioration, off-odor development, and significant reductions in shelf life [3]. Beyond spoilage organisms, foodborne pathogens, including *Escherichia coli* (*E.coli*), *Staphylococcus aureus* (*S. aureus*), and *Listeria monocytogenes* (*L. monocytogenes*), frequently co-contaminate fresh produce during harvesting, handling, and distribution, posing severe risks to consumer safety. Foodborne illnesses pose a significant global public health challenge, with an estimated 600 million people, nearly 1 in 10 worldwide, falling ill after consuming contaminated food annually. In the United States alone, approximately 48 million people suffer from foodborne illnesses each year, leading to 128,000 hospitalizations and 3000 deaths [4]. In China, the burden of foodborne diseases presents unique challenges within the global context. During 2003–2017, a total of 19,517 foodborne disease outbreaks were reported through the national surveillance system, resulting in 235,754 cases, 107,470 hospitalizations, and 1457 deaths, with bacterial pathogens accounting for 34.78% of confirmed outbreaks [5]. Between 2010 and 2017, fresh produce was linked to 12.7% of foodborne outbreaks with confirmed etiology in the United States, with 37.3% classified as multistate outbreaks resulting in 4748 illnesses, 1190 hospitalizations, and 55 deaths [6]. *Agaricus bisporus* is highly susceptible to microbial deterioration, with *Pseudomonas* spp. being the most frequently implicated spoilage microorganisms, responsible for characteristic browning, tissue softening, and off-odor development during post-harvest storage. Nevertheless, pathogenic microorganisms, including *E. coli*, *S. aureus*, and *L. monocytogenes*, have also been reported in association with fresh mushroom products, posing significant food safety risks that equally merit scientific investigation [7]. The broad spectrum of microbial threats associated with *Agaricus bisporus*, ranging from surface spoilage bacteria such as *Pseudomonas* spp. to dangerous pathogens including *E. coli*, *S. aureus*, and *L. monocytogenes*, underscores the need for packaging solutions capable of delivering sustained, broad-spectrum antimicrobial activity directly at the produce surface. Fresh *Agaricus bisporus* mushrooms are documented as highly susceptible to contamination by foodborne pathogens, including *L. monocytogenes*, *S. aureus*, and *E. coli*, across the entire production and processing chain, with retail surveys linking such contamination to severe infections, hospitalizations, and deaths globally [8,9]. In response, the U.S. FDA has classified mushroom-associated *L. monocytogenes* recalls as Class I, its highest risk designation, and under the Food Safety Modernization Act (FSMA, 21 CFR Part 112 & 117), mandates environmental monitoring for key pathogens across all mushroom production and processing facilities [10,11]. Given this global crisis of bacterial contamination threatening food safety worldwide, there is an urgent need for advanced antimicrobial interventions capable of addressing diverse bacterial threats across the food supply chain.

Nanotechnology has emerged as a promising field that enables the design of advanced antimicrobial substances with improved efficacy compared to conventional approaches [12]. Among various nanomaterials under investigation, gold nanoparticles (AuNPs) have attracted particular attention as a technological platform for food safety applications. AuNPs provide several distinct advantages, including broad-spectrum antimicrobial activity against both Gram-positive and Gram-negative bacteria, excellent biocompatibility with minimal toxicity concerns for human consumption, and tunable physicochemical properties that enable targeted functionality [13]. Studies have demonstrated that AuNPs exhibit broad-spectrum antimicrobial activity against common foodborne spoilage microorganisms and pathogens such as *Pseudomonas aeruginosa*, *E. coli*, *S. aureus*, *Salmonella typhimurium*, and *L. monocytogenes*, with antibacterial efficacy being size-dependent, where smaller nanoparticles (5–20 nm) generally show enhanced activity [14,15]. However, the antimicrobial efficacy and biocompatibility of AuNPs are critically dependent on the synthesis method employed [16]. The green synthesis approach using polyphenols as a reducer has gained attraction due to its being an environmentally benign, cost-effective, and sustainable alternative that aligns with the principles of green chemistry [17].

Cyclodextrin metal–organic frameworks (CD-MOFs) are highly crystalline porous structures assembled from cyclodextrins, which are biocompatible, water-soluble starch-derived oligosaccharides featuring hydrophobic internal cavities and hydrophilic external surfaces, with exceptional surface area and tunable dimensions well-suited for guest molecule encapsulation [18]. Although the use of CD-MOFs in the green synthesis of AuNPs remains limited, existing evidence suggests they are promising candidates for size-controlled synthesis, offering a sustainable alternative to conventional chemical stabilizers [19]. However, the practical deployment of CD-MOFs is significantly hindered by their inherent moisture instability, attributed to the hydroxyl groups on their exterior surfaces, limiting their applicability in aqueous environments, particularly impacting the prolonged antimicrobial effect of the AuNPs [20]. Therefore, to enhance CD-MOF water stability, crosslinking is a powerful post-synthetic modification strategy that creates additional covalent or coordination linkages within the framework structure. Furukawa et al. [21] first reported the fabrication of cubic gel particles with controlled nano-to-micrometer sizes by crosslinking cyclodextrin metal–organic frameworks (CD-MOFs). The resulting material exhibited enhanced water resistance, enabling its application under humid conditions. In the context of food packaging, CD-MOFs have emerged as multifunctional nanofillers capable of simultaneously improving the mechanical, barrier, and antimicrobial properties of biopolymer-based films. Their edible, renewable, and biodegradable nature, combined with high specific surface area and controllable porosity, makes them particularly attractive for the development of active packaging systems that meet both food safety and sustainability requirements. By serving as carriers for natural antimicrobial agents such as polyphenols, essential oils, and metal nanoparticles, CD-MOFs enable controlled and sustained release of bioactive compounds directly at the food surface, thereby extending shelf life while minimizing the use of synthetic preservatives [22]. For instance, β-CD-MOF loaded with carvacrol and incorporated into zein films demonstrated humidity-triggered sustained release behavior, achieving 96.3% release at 100% RH while retaining effective fruit preservation over extended storage [23]. These studies collectively highlight CD-MOFs as versatile platforms for designing stimuli-responsive antimicrobial packaging systems tailored to the specific microbial and environmental challenges of fresh produce preservation.

Polylactic acid-Zein composite films have emerged as promising materials for food packaging applications due to their complementary properties. PLA provides mechanical strength and thermoplastic processability essential for industrial-scale film fabrication, while zein contributes superior oxygen barrier performance critical for food preservation [24]. This synergistic combination yields a biodegradable packaging material derived from renewable resources, addressing both environmental sustainability and food safety requirements. Also, the antimicrobial properties and preservation efficacy of PLA-Zein composite films for fresh produce have been extensively investigated in recent years. For instance, Liu et al. [25] reported a PLA-Zein film exhibiting favorable packaging properties, antimicrobial activity, and effective maintenance of blueberry quality during storage. However, studies on the incorporation of CD-MOF-based materials into PLA-Zein composite films remain extremely scarce. More critically, to the best of our knowledge, no study has yet explored the integration of crosslinked-AuNPs@CD-MOF nanocomposites into PLA-Zein matrices, leaving their effects on film-forming behavior, water stability, and multifunctional performance entirely unexplored. This gap underscores the novelty of the present work. The most closely related prior work is Liang et al. [26], who fabricated an AgNPs@γ-CD-MOFs/PLA film achieving >80% inhibition of *E. coli*, *S. aureus*, and *Pseudomonas tolaasii*, and effective suppression of *Agaricus bisporus* browning over 12 days. While the present study shares the same application target and CD-MOF-based nanocomposite concept, several fundamental distinctions define its novelty. Unlike the AgNPs produced via in situ alkaline reduction of Ag^+^ within the CD-MOF framework in that study, AuNPs were synthesized here through targeted reduction of HAuCl_4_ using p-coumaric acid as a natural polyphenolic reducing agent, representing a distinct green synthesis strategy. More critically, Liang et al. employed unmodified γ-CD-MOFs, whose moisture sensitivity may result in burst nanoparticle release. In contrast, the crosslinking of the CD-MOF framework in the present study could transform this burst release into sustained AuNPs release. The film matrix further distinguishes both works, as the incorporation of zein into the PLA matrix introduces an additional protein-based barrier layer that may enhance mechanical properties and oxygen barrier performance compared to the PLA-only system [27].

This study aims to develop and optimize a crosslinked AuNPs@CD-MOF/PLA-Zein composite film, structural characterization, and functional evaluation, with the ultimate goal of establishing an effective active packaging system for the preservation of *Agaricus bisporus* against foodborne pathogens. In the context of food safety and regulatory compliance, it is important to emphasize that components employed in the fabrication of this system, including γ-cyclodextrin, p-coumaric acid as a natural reducing agent, zein as a plant-derived protein, and polylactic acid as a biodegradable polymer, are food-grade materials that are biocompatible, non-toxic, and widely recognized as suitable for food contact applications, thereby supporting the practical feasibility and safety of the proposed active packaging system. Specifically, AuNPs were synthesized via a green approach using CD-MOF as a stabilizer and p-coumaric acid (p-CA) as a natural reducing agent for the reduction of HAuCl_4_. The obtained material was crosslinked using Diphenyl Carbonate (DPC) through process optimization. We hypothesized that increasing the crosslinking density of AuNPs@CD-MOF via DPC would progressively restrict AuNPs release kinetics, thereby prolonging sustained antimicrobial activity, translating into enhanced preservation properties of the resulting CL-AuNPs@CD-MOF/PLA-Zein composite film. Comprehensive characterization was performed to investigate the structural properties of the crosslinked material, while its water stability was assessed through release studies, followed by evaluation of its antibacterial activity against *E. coli* and *S. aureus*, demonstrating a sustained long-term antibacterial effect compared to the non-crosslinked material. Subsequently, a PLA-Zein composite film was prepared and optimized based on the zein:PLA ratio and CL-AuNPs@CD-MOF content. The resulting film was thoroughly characterized, including its barrier properties, and its efficacy was evaluated for the preservation of *Agaricus bisporus* contaminated with *E. coli*, *S. aureus*, and *L. monocytogenes*.

## 2. Material and Methods

### 2.1. Materials

#### 2.1.1. Chemical Reagents

The γ-CD (98%, CAS number 1746-86-0), potassium hydroxide (KOH, 90%, CAS number 1310-58-3), Diphenyl carbonate (DPC, CAS: 102-09-0), and Polylactic acid (PLA, CAS: 26100-51-6, grade 4032D, Mw ~200 kDa, melt flow index 7 g/10 min at 210 °C/2.16 kg) pellets were obtained from Shanghai Macklin Biochemical Co., Ltd. (Shanghai, China). p-Coumaric Acid (CAS number 501-98-4) and Polyethylene glycol 400 (PEG 400, CAS: 25322-68-3) were obtained from Shanghai Yuanye Bio-Technology Co., Ltd. (Shanghai, China). Polyethylene glycol 8000 (PEG 8000, CAS: 25322-68-3) was purchased from Aladdin Bio-Chem Technology Co., Ltd. (Shanghai, China). Methanol (MeOH, 99.5%, CAS: 67-56-1), Ethanol (EtOH, 99.5%, CAS number 64-17-5), chloroauric acid (47.8%, CAS: 16903-35-8), N,N-dimethylformamide (DMF, CAS: 68-12-2), Triethylamine (TEA, CAS: 121-44-8), and Trichloromethane (chloroform, CAS: 67-66-3) were obtained from Sinopharm Chemical Reagents Co., Ltd. (Shanghai, China). Zein (CAS: 9010-66-6, purity ≥ 98%, Mw ~40 kDa, nitrogen content ≥ 16%) was obtained from Shanghai Bomei Chemicals Technology Co., Ltd. (Shanghai, China). Ultra-pure water was produced using a Smart-N purification system (Heal Force Biotech, Hong Kong, China). Phosphate Buffer Saline (PBS) was purchased from Beijing Solarbio Science & Technology Co., Ltd. (Beijing, China).

#### 2.1.2. Microbial Strains and Culture Media

LB Broth, LB Nutrient Agar, *Staphylococcus aureus* (ATCC 25923), *Escherichia coli* O157:H7 (ATCC 35150), and *Listeria monocytogenes* were purchased from Qingdao Hope Bio-technology Co., Ltd. (Qingdao, China). Airway smooth muscle cells (ASMCs) were purchased from the Cell Resource Center, Shanghai Institutes for Biological Sciences (Shanghai, China). Dulbecco’s modified Eagle medium (DMEM) high sugar medium was obtained from Shanghai Institute of Biological Sciences (Shanghai, China). Fetal bovine serum was obtained from Shanghai Yuanpei Biotechnology Co., Ltd. (Shanghai, China).

### 2.2. Methods

#### 2.2.1. Preparation of CD-MOF and CL-AuNPs@CD-MOF

CD-MOF was prepared following a modified version of previously reported methods [28,29,30]. Briefly, γ-CD (2962 mg) and KOH (1024 mg) were dissolved in ultrapure water (80 mL) under stirring at room temperature, then passed through a 0.45 μm membrane. The filtrate was mixed with MeOH (48 mL) in a conical flask and heated in a water bath (90 °C, 400 rpm) for 5 min. PEG 8000 (1024 mg) was then added, and stirring continued for 5 min before the flask was transferred to a cold-water bath and left overnight to allow white precipitate formation. The solid was collected by centrifugation (5000 rpm, 5 min), washed three times with methanol, resuspended in methanol, and dried under vacuum at 60 °C for 5 h to obtain the final CD-MOF powder.

To prepare CL-AuNPs@CD-MOF, CD-MOF (50 mg) was first suspended in ethanol (5 mL) containing p-coumaric acid (5 mg) and shaken at 400 rpm for 12 h at room temperature. The encapsulated composite was recovered by centrifugation (5000 rpm, 5 min) and dried under vacuum at 60 °C for 6 h. The dried composite (50 mg) was then resuspended in ethanol (5 mL) containing 1 mM chloroauric acid and incubated at 37 °C for 18 h at 180 rpm in the dark. The resulting AuNPs@CD-MOF was collected by centrifugation (5000 rpm, 5 min) [31,32]. For crosslinking, AuNPs@CD-MOF and diphenyl carbonate (DPC) at a 1:1 mass ratio were dispersed in DMF (20 mL) under magnetic stirring at 80 °C, followed by the addition of triethylamine (450 μL). The reaction was maintained at 80 °C for 18 h, then cooled to room temperature and quenched with 10 mL of 95% ethanol. The precipitate was washed twice with 10 mL of ethanol and then 10 mL of deionized water, then dried under vacuum at 60 °C for 6 h [33].

To optimize crosslinking conditions, three parameters were systematically investigated. First, the CD-MOF:DPC mass ratio was varied (1:2, 1:1.5, 1:1, 2:1, 3:1, and 6:1). Second, reaction time was evaluated at a fixed 1:1 ratio in DMF (20 mL) with TEA (450 μL) at 80 °C for 60, 180, 360, 720, 1080, and 1440 min. Third, reaction temperature was assessed at 20, 40, 60, 80, and 100 °C for 1080 min under otherwise identical conditions. After each reaction, the mixture was cooled, quenched with 10 mL 95% ethanol, washed twice with 10 mL of ethanol and 10 mL deionized water, and dried under vacuum at 60 °C for 6 h.

#### 2.2.2. Characterizations of CD-MOF and CL-AuNPs@CD-MOF

CD-MOF and CL-AuNPs@CD-MOF were characterized using the following instruments. Morphological characterization of MOF crystals was conducted using a scanning electron microscope (SEM) (Gemini SEM 360, ZEISS, Oberkochen, Germany) at an accelerating voltage of 3 keV. Before imaging, samples were fixed on conductive tape and sputter-coated with platinum. Transmission electron microscopy (TEM) (JEM-1230, JEOL, Akishima, Japan) was performed on freshly prepared specimens to assess crystal morphology; samples were dispersed in ethanol by ultrasonication using a digital water bath ultrasonicator (BJUCDTH30, Beijing Ultrasonic, Beijing, China) operating at 40 kHz for 10 min at 25 °C and deposited on copper grids before analysis. FTIR spectra of CD-MOF crystals and composite materials were acquired using a Nicolet IS50 spectrometer (Thermo Fisher Scientific, Waltham, MA, USA) over 500–4000 cm^−1^ at 4 cm^−1^ resolution, with samples ground and blended with KBr before pellet preparation. Surface chemical composition and binding states were characterized by X-ray photoelectron spectroscopy (XPS) using an AXIS SUPRA+ instrument (Kratos Analytical, Manchester, UK). Crystalline structures were analyzed by powder X-ray diffraction (PXRD) on a D8 Advance diffractometer (Bruker AXS, Karlsruhe, Germany) at 40 kV and 40 mA, scanning from 2° to 90° (2θ) with a step size of 0.02° and a dwell time of 0.1 s per step. Theoretical diffraction patterns of CD-MOF were generated using Materials Studio 2017 (Cambridge Crystallographic Data Centre, Cambridge, UK). Thermogravimetric analysis (TGA) of CD-MOF and CL-AuNPs@CD-MOF was carried out on a STARe System TGA2 (Mettler Toledo, Greifensee, Switzerland). Approximately 7 mg of each sample was placed in an aluminum pan and heated from 30 to 750 °C at 10 °C/min under a nitrogen atmosphere (50 mL/min).

#### 2.2.3. Evaluation of Crosslinking Performance

The crosslinking yield was determined gravimetrically by recording the mass of AuNPs@CD-MOF before (*M*_0_) and after (*M_f_*) the reaction. The product was washed with ethanol and deionized water to remove unreacted reagents, then dried under vacuum at 60 °C for 6 h [34]. Yield was calculated as

(1) $$Yield(\%)=\frac{M_{f}}{M_{0}}\times 100$$

Gold ion release was assessed by dispersing 50 mg of CL-AuNPs@CD-MOF in 50 mL of deionized water under magnetic stirring at 50 rpm. Aliquots of 200 μL were collected at 1, 3, and 7 days, replaced with equal volumes of fresh water to maintain constant volume, and digested with aqua regia (HCl/HNO_3_, 3:1) before 1000-fold dilution. Gold ion concentration was quantified by ICP-MS (NexION 300X, PerkinElmer, Waltham, MA, USA), and cumulative release rate was calculated as

(2) $$R\left(\%\right)=\frac{M_{Au}}{M}\times 100\%$$

where R represents the cumulative release rate (%), *M_Au_* is the gold ion content (mg), and M indicates the total mass (mg) of CL-AuNPs@CD-MOF material. All measurements were performed in triplicate for each sample.

#### 2.2.4. Stability Study of CL-AuNPs@CD-MOF in Water

To compare gold ion release kinetics, 50 mg each of CL-AuNPs@CD-MOF and non-crosslinked Au-NPs@CD-MOF were dispersed in 50 mL of deionized water at 50 rpm. Aliquots of 200 μL were withdrawn at predetermined intervals (0–12,000 min), replaced with fresh water, and analyzed by ICP-MS. Cumulative release (%) was calculated using Equation (2). Four mathematical models were applied to the gold ion release in water results: the zero-order kinetic model, the first-order kinetic model, the Higuchi model, and the Korsmeyer–Peppas model. The corresponding kinetic equations are presented in Table 1. 

In addition, the UV-Vis absorbance spectra of AuNPs@CD-MOF and CL-AuNPs@CD-MOF were recorded using a UV-Vis spectrophotometer (UV-2550, Shimadzu Corporation, Kyoto, Japan) over a wavelength range of 200–800 nm.

#### 2.2.5. Biocompatibility Analysis of CL-AuNPs@CD-MOF

Cell viability was assessed following Guo et al. [28]. Airway smooth muscle cells (ASMCs) were cultured in DMEM supplemented with 10% fetal bovine serum and 1% penicillin-streptomycin at 37 °C. CL-AuNPs@CD-MOF were prepared in DMEM at concentrations of 62.5, 125, 250, 500, and 1000 μg/mL. After 10 μL of each concentration was added to wells and incubated for 24 h at 37 °C, 10 μL of CCK-8 solution (1×) was introduced and incubated for a further 4 h. Absorbance was recorded at 450 nm, and cell viability (%) was calculated as

(3) $$A(\%)=(A_{s}/A_{c})\times 100\%$$

where *A_s_* and *A_c_* denote the absorbance values of the treatment group and control group, respectively. All measurements were performed in triplicate.

#### 2.2.6. Preparation of CL-AuNPs@CD-MOF/PLA-Zein Composite Films

PLA-Zein blend films were prepared by solution casting using a trichloromethane/methanol co-solvent (4:1 *v*/*v*) [35]. Briefly, trichloromethane (20 mL) and methanol (5 mL) were mixed, and zein (1.5 g) was added under stirring at 500 rpm for 10 min. PLA (1.5 g) was then gradually incorporated, followed by PEG 400 (40% *w*/*w* of total polymer mass) acting as a compatibilizer and plasticizer. The mixture was stirred for 3 h until complete dissolution. The resulting solution was cast onto a PTFE substrate and dried at room temperature until full solvent evaporation, yielding a uniform film.

The PLA-Zein film formulation was optimized following an adapted method described by Huang et al. [35] by varying the zein to PLA mass ratios (1:2, 1:1, and 2:1) and PEG 400 content (20% and 40% *w*/*w*, based on total polymer weight). Sample nomenclature was designated as follows: PG_20% represented pure PLA with 20% PEG, while ZPG_1:2_20%, ZPG_1:1_20%, and ZPG_2:1_20% denoted PLA-Zein blends at mass ratios of 1:2, 1:1, and 2:1, respectively, each containing 20% PEG. Similarly, PG_40% represented pure PLA with 40% PEG, while PLA-Zein samples with 40% PEG were designated as ZPG_1:2_40%, ZPG_1:1_40%, and ZPG_2:1_40%.

CL-AuNPs@CD-MOF was incorporated into the PLA-Zein films following an adapted procedure from Guo et al. [28]. After dissolving all film components as previously described, predetermined amounts of CL-AuNPs@CD-MOF powder (50, 100, 150, and 200 mg; corresponding to 3.13, 6.25, 9.38, and 12.50 wt.% relative to the total polymer content) were weighed and added to the homogeneous polymer solution. The mixture was stirred for 30 min to ensure a uniform dispersion of the nanoparticles before being cast onto a flat polytetrafluoroethylene (PTFE) substrate. The films were then allowed to dry in a ventilated area at room temperature until complete solvent evaporation occurred.

#### 2.2.7. Characterization of the Composite Films

The surface and cross-sectional morphologies of PLA-Zein and CL-AuNPs@CD-MOF/PLA-Zein composite films were characterized using scanning electron microscopy (SEM, Gemini SEM 360, ZEISS, Oberkochen, Germany). For surface morphology analysis, film samples were cut into approximately 1 cm × 1 cm pieces using a scalpel and mounted on aluminum stubs with conductive adhesive. For cross-sectional analysis, films were immersed in liquid nitrogen, then fractured using pre-cooled tweezers to obtain clean cross-sections. All samples were observed at an accelerating voltage of 3 keV [27]. A scanning electron microscope (SEM) (Gemini SEM 360, ZEISS, Oberkochen, Germany) was used to observe the surface and the cross-section of the composite films. A Fourier transform infrared spectrometer (FTIR) (Nicolet IS50, Thermo Fisher, USA) over 500–4000 cm^−1^ at 4 cm^−1^ resolution, with samples ground and blended with KBr before pellet preparation, was used to collect spectra of the composite films. A Powder X-ray diffraction (PXRD) (D8 Advance, Bruker AXS, Karlsruhe, Germany) at 40 kV and 40 mA, scanning from 2° to 90° (2θ) with a step size of 0.02° and a dwell time of 0.1 s per step, was used to analyze the diffraction patterns of the composite films.

Mechanical properties of the composite films were evaluated using a universal testing machine (ZwickRoell, Ulm, Germany) fitted with a 500 N load cell, at a crosshead speed of 1.5 mm/min and a preload of 0.05 N. Dumbbell-shaped specimens were prepared following ASTM D882. Tensile strength (TS), elongation at break (EAB), and elastic modulus were recorded at the point of fracture for both PLA-Zein and CL-AuNPs@CD-MOF/PLA-Zein films. All samples were conditioned at constant temperature and humidity for 48 h before testing, and five replicates per group were averaged. TS was calculated using the following equation:

(4) $$TS=\frac{F}{S}$$

where TS is the tensile strength (MPa), F is the maximum tension (N) that the composite film can withstand when it breaks, and S is the cross-sectional area (m^2^) of the composite film.

The elongation at break of the PLA-Zein and CL-AuNPs@CD-MOF/PLA-Zein composite films was calculated using the following equation:

(5) $$ELO=\frac{L-L_{o}}{L_{o}}\times 100\%$$

where *ELO* is the elongation at break (%) of the composite film, *L_o_* is the initial length (mm) of the composite film, and *L* is the length (mm) of the composite film at break.

The force–displacement curve of the composite film was converted into a stress–strain curve. The tensile elastic modulus (Young’s modulus) of the composite film was determined from the slope of the linear region of the stress–strain curve and calculated using the following equation:

(6) $$E=\frac{\sigma }{\varepsilon }$$

where *E* is the tensile elastic modulus (MPa) of the composite film, *σ* is the stress (MPa), and *ε* is the strain.

#### 2.2.8. Barrier Properties of CL-AuNPs@CD-MOF/PLA-Zein Composite Films

The contact angle and surface tension were measured using a DropMeter A-100P contact angle goniometer (Haishu Maishi Testing Technology Co., Ltd., Ningbo, China). The instrument has a minimum droplet resolution of 0.5 μL, and a droplet volume of 6 μL was used for static contact angle measurements.

Water vapor permeability (WVP) of PLA-Zein and CL-AuNPs@CD-MOF/PLA-Zein films was measured following GB/T 1037-2021 using the desiccant method [36]. Film samples were sealed over aluminum cups containing ~10 g of anhydrous CaCl_2_, then placed in a climate chamber at 38 ± 0.5 °C and 90 ± 2% RH. Cup mass was recorded every 24 h over 7 days, and the water vapor transmission rate (WVTR) was calculated as

(7) $$WVTR=\frac{\Delta m}{\left(t\times A\right)}$$

where Δm is the mass gain (g), t is the testing time (d), and A is the effective permeation area (m^2^).

Oxygen permeability was measured following GB/T 1038.1-2022using a differential pressure method [37]. Film specimens were mounted in the test chamber at 23 ± 0.5 °C and 0% RH, with pure oxygen (99.9%) applied to one side at atmospheric pressure while the permeate side was kept under vacuum. The oxygen transmission rate (OTR) was recorded at steady state and calculated as

(8) $$OTR=\frac{V}{\left(A\times t\times \Delta P\right)}$$

where V is the volume of oxygen permeating through the film (cm^3^), A is the effective permeation area (m^2^), t is the testing time (24 h), and ΔP is the pressure difference across the film (0.1 MPa).

#### 2.2.9. Release Study of Gold from CL-AuNPs@CD-MOF/PLA-Zein Composite Film

CL-AuNPs@CD-MOF/PLA-Zein composite films (1 cm diameter) were immersed in 20 mL of ethanol aqueous solutions at varying concentrations (0, 25, 50, 75, and 100% *v*/*v*) at room temperature to simulate environments with different moisture content levels, thereby evaluating the humidity-responsive controlled release behavior of the composite films [38]. At predetermined time intervals (60, 240, 720, 1440, 2880, 4320, and 5760 min), aliquots of 200 μL were withdrawn from the release medium and replaced with an equal volume of fresh medium. The cumulative release percentage was calculated using the following Equation (2).

Where R was the cumulative release percentage (%), *M_Au_* was the cumulative release amount of gold ions (mg), and M was the total amount of gold ions embedded in the CL-AuNPs@CD-MOF/PLA-Zein composite film.

#### 2.2.10. In Vitro Antibacterial Study of CL-AuNPs@CD-MOF and CL-AuNPs@CD-MOF/PLA-Zein Composite Film

Long-term antimicrobial activity of CL-AuNPs@CD-MOF was evaluated against *S. aureus* ATCC 25923 and *E. coli* O157:H7. Cultures grown in LB broth at 37 °C (180 rpm, 18 h) were adjusted to 1 × 10^6^ CFU/mL in sterile PBS. Bacterial suspensions (100 μL) were combined with 100 μL of LB broth containing CL-AuNPs@CD-MOF or Au-NPs@CD-MOF in 96-well plates at concentrations of 115 μg/mL for *E. coli* and 50 μg/mL for *S. aureus*, selected based on MIC and MBC values reported for comparable nanoparticle systems [39,40,41]. Bacteria without MOF and LB broth alone served as positive and negative controls, respectively. Plates were incubated at 37 °C, and viable counts were determined by plate counting at days 0–7 [42].

The in vitro antibacterial efficacy of CL-AuNPs@CD-MOF/PLA-Zein composite films and PLA-Zein films was evaluated against *S. aureus* ATCC 25923, *E. coli* O157:H7, and *L. monocytogenes*. Bacterial suspensions were prepared and adjusted to 1 × 10^6^ CFU/mL in sterile PBS. Film samples (3 cm^2^) were immersed in 5 mL of each bacterial suspension and incubated at 37 °C under continuous agitation at 180 rpm for 48 h. Viable bacterial counts were determined at predetermined time intervals (0, 8, 16, 24, 32, 36, and 48 h) using the plate counting method. All experiments were performed in triplicate to ensure reproducibility.

#### 2.2.11. Preservation Study of CL-AuNPs@CD-MOF/PLA-Zein Composite Film on *Agaricus bisporus*

Fresh *Agaricus bisporus* were purchased from a local market and stored at 4 °C. Mushrooms of uniform size and free of visible damage were individually inoculated by spreading 50 μL of bacterial suspension (10^6^ CFU/mL) of *S. aureus* ATCC 25923, *E. coli* O157:H7, or *L. monocytogenes* over the cap surface using a sterile spreader, then left to dry under aseptic conditions for 30 min. Inoculated mushrooms were divided into three groups: (1) wrapped with CL-AuNPs@CD-MOF/PLA-Zein composite film, (2) wrapped with pristine PLA-Zein film (positive control), and (3) wrapped with commercial polyethylene (PE) film (negative control). Each mushroom was placed in a disposable polyethylene terephthalate (PET) tray, sealed, and stored at 4 °C and 90% RH to simulate refrigerated storage conditions. The preservation study was conducted over a 12-day storage period, with samples evaluated at designated time points (day 0, 3, 5, 7, 9, and 12) [24,39].

The weight of individual mushrooms from each treatment group was measured at predetermined time intervals using an analytical balance. Weight loss was calculated as a percentage of the initial weight using the following equation:

(9) $$Weight\;loss\left(\%\right)=\frac{W_{o}-W_{t}}{W_{o}}\times 100$$

where *W_o_* is the initial weight (g), and *W_t_* is the weight (g) at time *t*.

The color of mushroom caps was evaluated using a colorimeter (CR-400, Konica Minolta, Japan). L* (lightness, 0–100), a* (red-green axis), and b* (yellow-blue axis) values were recorded.

The total color difference (ΔE) of *Agaricus bisporus* mushrooms during storage was calculated according to the CIE L* a* b* color space using the following equation:

(10) $$\mathrm{Total}\;\mathrm{color}\;\mathrm{difference}\;(\Delta E)=\sqrt{{\left({\Delta L}^{*}\right)}^{2}+{\left({\Delta a}^{*}\right)}^{2}+{\left({\Delta b}^{*}\right)}^{2}}$$

where ΔL*, Δa*, and Δb* represent the differences in lightness, redness, and yellowness, respectively, between the sample at each storage time point and the initial measurement on Day 0 [24].

To determine bacterial counts on mushrooms, at predetermined time intervals (0, 3, 5, 7, 9, and 12 days), mushroom samples (5 g) from each treatment group were aseptically transferred to sterile stomacher bags containing 90 mL of sterile 0.1% (*w*/*v*) peptone water and homogenized for 2 min. Serial ten-fold dilutions were prepared using sterile peptone water, and 100 μL aliquots from appropriate dilutions were spread-plated onto Plate Count Agar (PCA) in triplicate.

**Figure 1:**
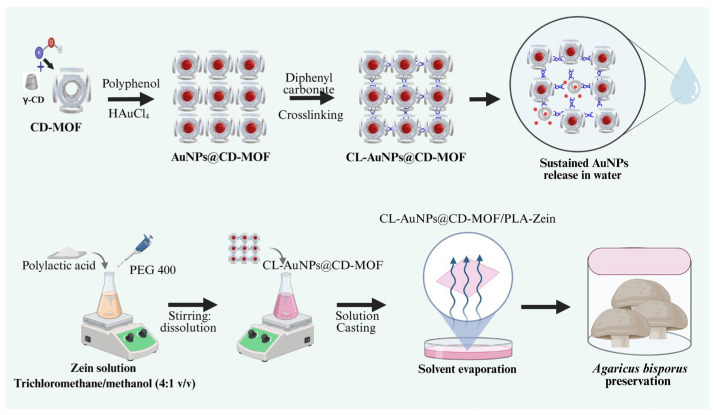
Schematic illustration of CL-AuNPs@CD-MOF/PLA-Zein synthesis for *Agaricus bisporus* preservation.

The schematic illustration of CL-AuNPs@CD-MOF/PLA-Zein synthesis for *Agaricus bisporus* preservation is presented in Figure 1.

#### 2.2.12. Data Analysis

Statistical analysis was performed using IBM SPSS 25.0 with one-way ANOVA and Tukey’s post hoc test (*p* < 0.05). All experiments were conducted in triplicate, and data were visualized using Origin 2018 and GraphPad Prism 9. The graphical abstract was created in BioRender (https://www.biorender.com/).

## 3. Results and Discussion

### 3.1. Characterizations of CD-MOF and CL-AuNPs@CD-MOF

**Figure 2:**
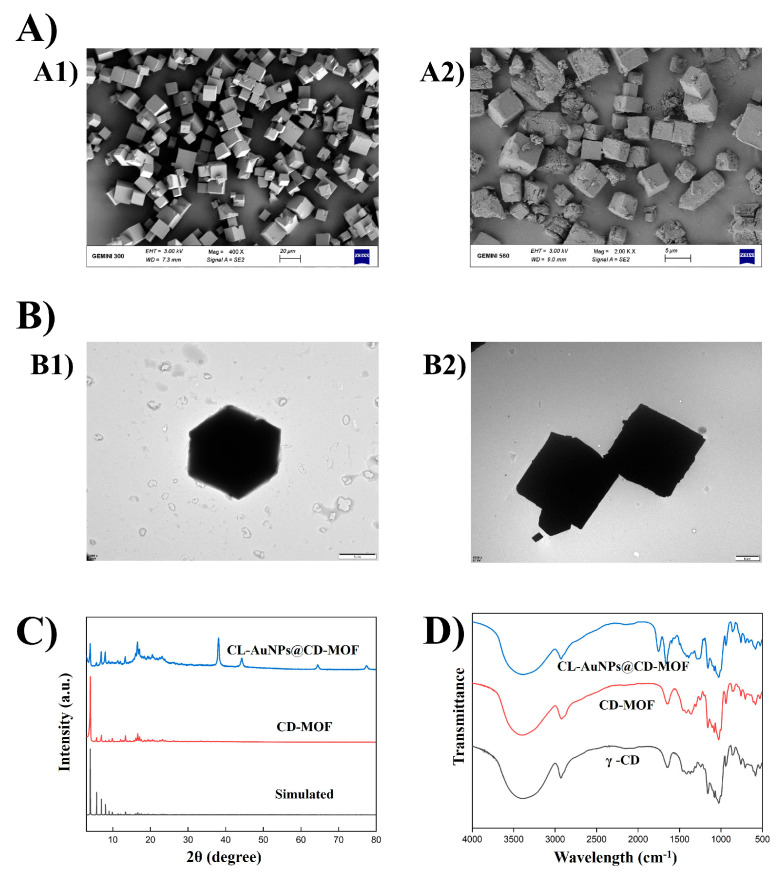
(**A**) SEM morphology images of [(**A1**)] CD-MOF, [(**A2**)] CL-AuNPs@CD-MOF; (**B**) TEM images of [(**B1**)] CD-MOF, [(**B2**)] CL-AuNPs@CD-MOF; (**C**) PXRD patterns of simulated CD-MOF, CD-MOF, CL-AuNPs@CD-MOF; (**D**) FTIR spectra of γ-CD, CD-MOF, CL-AuNPs@CD-MOF.

The SEM and TEM images in Figure 2A,B confirm the successful synthesis of CD-MOF. Following the preparation of CL-AuNPs@CD-MOF, the images show that the characteristic cubic morphology of CD-MOF was preserved, with only minor structural changes attributable to solvent effects, AuNP loading, and the crosslinking process [40]. TEM analysis revealed that CL-AuNPs@CD-MOF consisted of well-dispersed spherical particles with a mean diameter of 7.23 ± 2.09 nm and a coefficient of variation of 28.85%, indicating a relatively uniform size distribution (Figure S1). The PXRD in Figure 2C showed that CD-MOF presents well-defined crystalline diffraction peaks at 2θ angles of 4.0°, 5.7°, 6.9°, 13.4°, and 16.7°, which were in strong agreement with both simulated patterns and previously published data [43]. Following AuNP synthesis and crosslinking, the characteristic CD-MOF diffraction peaks at 2θ values of 4.0°, 5.7°, 6.9°, 13.4°, and 16.7° were retained but with notably reduced intensities, suggesting partial disruption of the CD-MOF crystalline structure during the crosslinking process [44]. Furthermore, the emergence of new diffraction peaks at 38.0°, 44.2°, 64.5°, and 77.4°, corresponding to the (111), (200), (220), and (311) crystallographic planes of metallic gold, confirmed the successful incorporation of AuNPs within the CD-MOF matrix [45]. The Fourier transform infrared (FTIR) spectra displayed in Figure 2D reveal that γ-CD and CD-MOF exhibited characteristic peaks at approximately 3500 cm^−1^ (O–H stretching from cyclodextrin hydroxyl groups), 2900 cm^−1^ (aliphatic C–H stretching), and a complex fingerprint region between 1000 and 1500 cm^−1^ attributed to C–O–C glycosidic bonds and C–OH groups of the cyclodextrin framework [46]. Following CL-AuNPs@CD-MOF synthesis, the FTIR spectrum confirmed successful crosslinking between cyclodextrin hydroxyl groups and diphenyl carbonate, as evidenced by a marked decrease in the –OH stretching band intensity around 3500 cm^−1^, indicating the consumption of free hydroxyl groups during the reaction [47]. Additionally, the emergence of an absorption peak around 1750 cm^−1^, characteristic of carbonate ester carbonyl stretching (–O–C(=O)–O–), confirmed the formation of covalent bridges between cyclodextrin molecules [48,49]. Subtle shifts in the fingerprint region (1200–900 cm^−1^) where C–O stretching vibrations characteristic of both the cyclodextrin glycosidic bonds and the newly formed carbonate ester linkages overlap further supported successful crosslinking while indicating that the fundamental CD-MOF structure was preserved throughout the modification process [50]. The XPS analysis Figure S2 provided additional insights into the molecular structure and elemental distribution of CL-AuNPs@CD-MOF. The C 1s spectrum shows three distinct carbon environments: C–C at 284.8 eV; C–O at 286.26 eV, reflecting the ether and hydroxyl groups of the cyclodextrin structure; and C=O at 287.67 eV, confirming the formation of ester carbonyl species resulting from covalent crosslinking [51,52]. The K 2p peaks, observed at 295 eV and 292 eV, indicate potassium coordination within the MOF framework in CL-AuNPs@CD-MOF [53]. Additionally, Au 4f peaks at approximately 84.5 eV (Au 4f7/2) and 88.11 eV (Au 4f5/2) confirmed the presence of metallic gold (Au^0^). These XPS results confirm that crosslinking effectively transforms the CD-MOF framework through covalent ester bond formation while maintaining the structural integrity of the material and preserving the metallic state of gold nanoparticles (Au^0^). Thermogravimetric analysis (TGA) assessed the thermal behavior of CL-AuNPs@CD-MOF across 50–750 °C (Figure S3). All materials exhibited a comparable initial mass reduction of ~4.2% near 100 °C, attributed to moisture desorption and solvent evaporation. Substantial degradation occurred above 300 °C, with the derivative TGA (DTGA) analysis revealing a dominant decomposition peak at T~max~ = 295 °C for the CL-AuNPs@CD-MOF, and ~50% mass loss recorded across all samples; however, CL-AuNPs@CD-MOF showed an additional 6.9% loss at this stage. Beyond 360 °C, progressive decomposition continued similarly across all materials, reaching ~70% total mass loss. At 750 °C, CL-AuNPs@CD-MOF retained 4.7% more residual mass than CD-MOF, likely due to thermally stable carbonaceous char formed from the diphenyl carbonate crosslinker, which acts as a protective barrier against framework decomposition [54,55]. However, the improved thermal stability could also be partly attributed to the AuNPs content, since gold nanoparticles are inherently stable at high temperatures and decompose well beyond the thermal range of the CD-MOF framework. Furthermore, owing to the excellent thermal conductivity of gold, AuNPs can rapidly dissipate localized heat across the composite matrix, minimizing the formation of thermal hotspots that would otherwise accelerate the decomposition of the surrounding framework [56]. Overall, these findings indicate that AuNPs synthesis combined with crosslinking improved the thermal stability of the material [57].

### 3.2. Optimization of the Preparation of CL-AuNPs@CD-MOF and Evaluation of Crosslinking Performance

**Figure 3:**
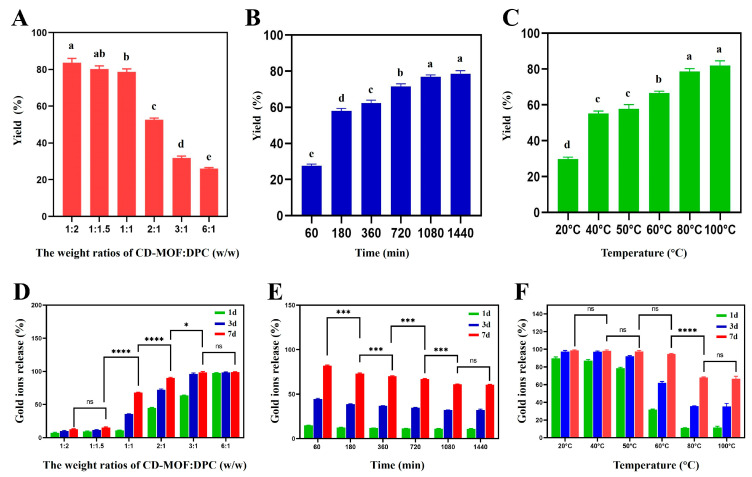
Optimization of the crosslinking process. The yield of CL-AuNPs@CD-MOF: (**A**) Effect of weight ratios of CD-MOF:DPC, (**B**) effect of reaction time, (**C**) effect of temperature. Gold ion release of CL-AuNPs@CD-MOF: (**D**) Effect of weight ratios of CD-MOF:DPC, (**E**) effect of reaction time, (**F**) effect of temperature. Values are expressed as mean ± SD. Different lowercase letters above bars indicate statistically significant differences among groups (one-way ANOVA with Tukey’s post-hoc test, *p* < 0.05); bars sharing the same letter are not significantly different. In the Gold ion release groups, statistical significance is indicated as ns (not significant) (*p* ≥ 0.05), * (*p* < 0.05), *** (*p* < 0.001), and **** (*p* < 0.0001) for CD-MOF:DPC ratio, reaction time, and temperature groups.

Yield was evaluated to quantify material recovery and structural integrity following crosslinking and water washing. Three parameters were optimized: CD-MOF to DPC weight ratio, reaction time, and temperature (Figure 3A–C). Regarding the CD-MOF:DPC ratio, yield decreased as CD-MOF content increased relative to DPC, from 83.70% at a 1:2 ratio to 53.61% at 2:1 and 31.70% at 6:1, indicating that insufficient crosslinking agent availability leads to incomplete network formation and reduced material recovery [58]. Reaction time significantly influenced yield, which rose from 27.59% at 60 min to 76.83% at 1080 min, beyond which no further increase was observed, reflecting the kinetics of hydroxyl group reaction with DPC until equilibrium was reached [59]. Similarly, temperature had a notable effect, with yield increasing from 29.75% at 20 °C to a maximum of 78.60% at 80 °C, after which no additional improvement was recorded. Elevated temperature enhances reactant diffusion and provides the activation energy needed for efficient crosslinking, though beyond 80 °C, all accessible reactive sites are consumed [60,61].

In Figure 3D–F, AuNPs release in water served as an indirect indicator of crosslinking efficiency, since inadequate crosslinking compromises the water stability of the CD-MOF framework, leading to structural collapse and AuNP leaching. The influence of the CD-MOF:DPC ratio on gold ion release was first examined. At a 2:1 ratio only 12.65% of gold was released after 7 days, while a 1:1 ratio yielded sustained release of 68.12% by day 7. At 1:2, rapid release was observed from day 1, and at 6:1, burst release reaching 97% occurred within the first day, indicating that higher CD-MOF:DPC ratios reduce crosslink density and facilitate rapid ion diffusion, while lower ratios produce tighter networks that retard release [62,63]. Regarding reaction time, gold release decreased progressively from 82% at 60 min to 67% at 1080 min, with no significant changes beyond this point, confirming complete network formation and slower ion diffusion with extended crosslinking [64]. Temperature had a pronounced effect on release behavior. Burst release of 89.60% within day 1 was observed at 20 °C, persisting up to 40 °C. At 60 °C, release dropped to 31.61% on day 1, while at 80 °C, the most controlled profile was achieved with only 11% on day 1 and 68% by day 7. Higher temperatures enhance crosslinking efficiency and network density, reducing pore size and restricting gold ion diffusion, with no further improvement beyond 80 °C [65].

Based on the optimization studies, the optimal conditions for synthesizing CL-AuNPs@CD-MOF that achieved both high yield and controlled gold ion release were: CD-MOF:DPC weight ratio of 1:1, reaction time of 1080 min, and reaction temperature of 80 °C.

### 3.3. Stability Study of CL-AuNPs@CD-MOF in Water

**Figure 4:**
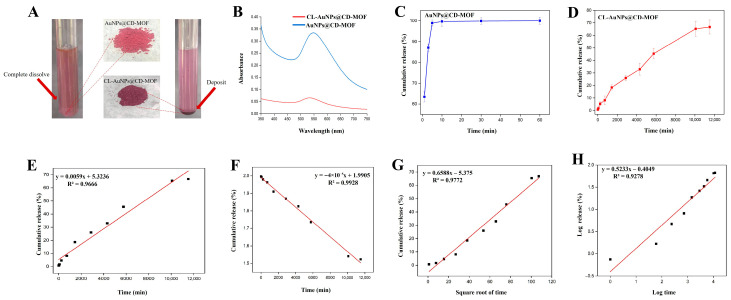
(**A**) Photographs of AuNPs@CD-MOF and CL-AuNPs@CD-MOF powder dispersed in water. (**B**) UV absorption curves of AuNPs@CD-MOF and CL-AuNPs@CD-MOF dispersed in water. (**C**) The release curve of AuNPs@CD-MOF in water. (**D**) The release curve of CL-AuNPs@CD-MOF in water. Fitting equations: the zero-order equation (**E**), the first-order equation (**F**), the Higuchi equation (**G**), and the Korsmeyer–Peppas equation (**H**).

Gold ion release experiments were conducted on both the prepared CL-AuNPs@CD-MOF and its non-crosslinked counterpart, AuNPs@CD-MOF. Figure 4A presents photographs of AuNPs@CD-MOF and CL-AuNPs@CD-MOF dispersed in water. After 60 min, AuNPs@CD-MOF had completely dissolved, whereas CL-AuNPs@CD-MOF remained as a visible precipitate in the tube [66]. The UV-vis spectra of both samples (Figure 4B) revealed that the surface plasmon resonance (SPR) peak intensity of AuNPs@CD-MOF was significantly higher than that of CL-AuNPs@CD-MOF, indicating reduced gold release from the crosslinked material compared to its non-crosslinked form. Furthermore, ICP-MS analysis was employed to quantify the time-dependent release profiles of gold (Figure 4C,D). Non-crosslinked AuNPs@CD-MOF exhibited burst release, with 63.46 ± 2.55% of gold released within 1 min and complete release after 10 min [67]. In contrast, CL-AuNPs@CD-MOF demonstrated sustained release, with only 1.82 ± 1.75% released at 60 min, rising to 18.39 ± 2.05% at 1440 min and 66.66 ± 5.54% at 11,520 min, confirming that crosslinking imparted water resistance and enabled controlled gold release [66].

Due to the burst release behavior of AuNPs@CD-MOF, only the release kinetics of CL-AuNPs@CD-MOF were analyzed. Four kinetic models were fitted to the experimental data (Figure 4E–H, Table 2). The correlation coefficients (R^2^) were compared to determine the best-fitting model. The first-order kinetic model showed the highest correlation (R^2^ = 0.9928), indicating the best fit, followed by the Higuchi model (R^2^ = 0.9772), the zero-order model (R^2^ = 0.9666), and the Korsmeyer–Peppas model (R^2^ = 0.9278). The comparable fit quality across these models indicates that the release mechanism likely involves multiple simultaneous processes rather than a single dominant mechanism. This release pattern is typical of diffusion-controlled systems and suggests that the crosslinked CD-MOF network effectively regulates gold ion release through a concentration-gradient-driven diffusion mechanism [68].

### 3.4. In Vitro Antibacterial Study of CL-AuNPs@CD-MOF

**Figure 5:**
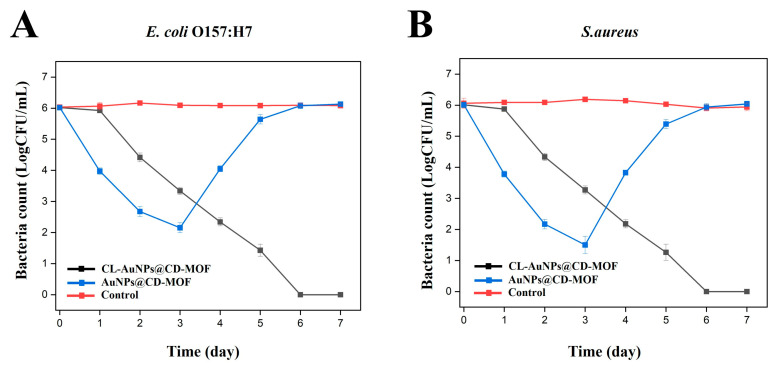
(**A**) Bacterial log reduction profiles of CL-AuNPs@CD-MOF and AuNPs@CD-MOF against *E. coli* O157:H7. (**B**) Bacterial log reduction profiles of CL-AuNPs@CD-MOF and AuNPs@CD-MOF against *S. aureus*.

Antibacterial assays of CL-AuNPs@CD-MOF and AuNPs@CD-MOF were conducted against *E. coli* and *S. aureus*; bacteria without treatment served as the control. After 3 days, bacterial counts with CL-AuNPs@CD-MOF were 3.34 and 3.27 log CFU/mL for *E. coli* and *S. aureus*, respectively, compared to lower counts of 2.15 and 1.50 log CFU/mL with AuNPs@CD-MOF, reflecting its burst release of a higher initial AuNPs dose. By day 4, however, CL-AuNPs@CD-MOF counts continued to decline to 2.34 and 2.18 log CFU/mL, while AuNPs@CD-MOF counts rebounded sharply to 4.05 and 3.82 log CFU/mL for *E. coli* and *S. aureus*, respectively (Figure 5). This reversal is attributed to the rapid depletion of AuNPs from AuNPs@CD-MOF, which allowed bacterial regrowth, whereas the sustained release from CL-AuNPs@CD-MOF maintained continuous antibacterial activity [69]. By day 7, CL-AuNPs@CD-MOF achieved near-complete bacterial elimination with counts of only 0.17 and 0.12 log CFU/mL for *E. coli* and *S. aureus*, while AuNPs@CD-MOF counts rose to 6.13 and 6.04 log CFU/mL, indicating complete loss of antibacterial activity [70]. These results confirm that crosslinking enables prolonged antimicrobial efficacy through controlled AuNPs release. 

In addition, the cell viability of airway smooth muscle cells (ASMCs) exposed to CL-AuNPs@CD-MOF was evaluated to determine its cytotoxic profile, with findings illustrated in Figure S5. Viability exceeded 90% across all tested concentrations, confirming a favorable safety profile. Notably, the diphenyl carbonate-based crosslinking treatment applied to improve aqueous stability did not compromise the inherent biocompatibility of the CD-MOF scaffold, supporting the suitability of CL-AuNPs@CD-MOF for antimicrobial and biomedical applications [71]. 

### 3.5. Characterizations CL-AuNPs@CD-MOF/PLA-Zein Composite Films

**Figure 6:**
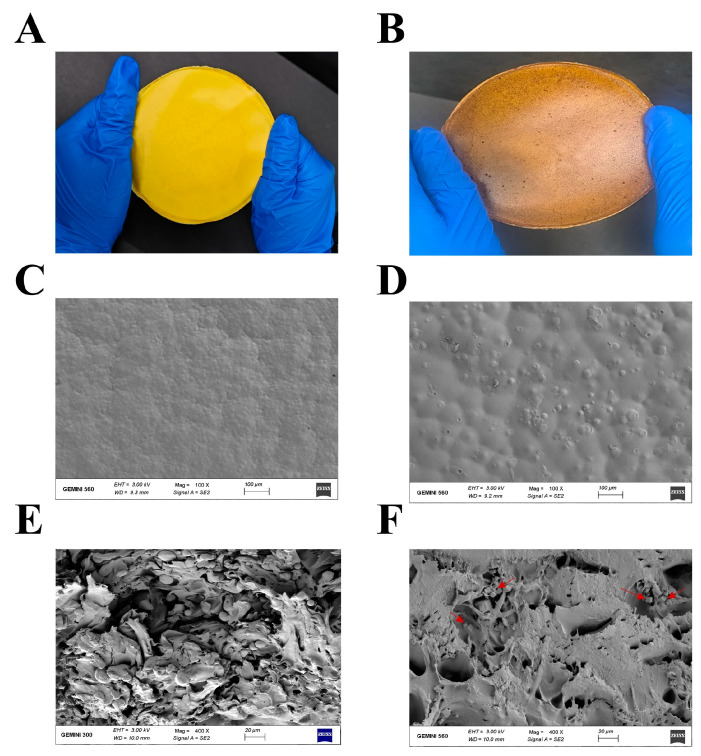
(**A**) Photograph of PLA-Zein composite film. (**B**) Photograph of CL-AuNPs@CD-MOF/PLA-Zein composite film. (**C**) SEM image of the surface of PLA-Zein composite film. (**D**) SEM image of the surface of CL-AuNPs@CD-MOF/PLA-Zein composite film. (**E**) SEM image of the cross-section of PLA-Zein composite film. (**F**) SEM image of the cross-section of CL-AuNPs@CD-MOF/PLA-Zein composite film with red arrows showing CL-AuNPs@CD-MOF particles.

The PLA-Zein composite film (Figure 6A) was first prepared, then CL-AuNPs@CD-MOF was incorporated to fabricate the CL-AuNPs@CD-MOF/PLA-Zein composite film (Figure 6B). SEM analysis revealed a slightly roughened surface for the PLA-Zein composite film (Figure 6C). Upon incorporation of CL-AuNPs@CD-MOF, the resulting CL-AuNPs@CD-MOF/PLA-Zein composite film displayed increased surface irregularity, which can be attributed to the presence of CL-AuNPs@CD-MOF particles, the heterogeneous nature of the polymer blend, and the surface morphology typically associated with the solvent-casting process (Figure 6D) [72]. Cross-sectional SEM images revealed a microarchitectured structure in the PLA-Zein composite film (Figure 6E). The CL-AuNPs@CD-MOF/PLA-Zein composite film showed CL-AuNPs@CD-MOF particles well-distributed within the PLA-Zein matrix, maintaining their structural integrity throughout the film formation process (Figure 6F) [73]. Overall, the SEM analysis confirmed the successful incorporation of CL-AuNPs@CD-MOF into the PLA-Zein composite matrix with uniform particle distribution and preserved MOF structural integrity, while the phase-separated morphology characterized by dispersed zein spherical domains within a continuous PLA matrix is consistent with the known immiscibility of both polymers. The dispersed zein domains are proposed to enhance oxygen barrier performance through the creation of tortuous diffusion pathways for gas molecules at the PLA-Zein interface, and the intrinsic barrier contribution of zein’s compact α-helical secondary structure, stabilized by extensive intramolecular hydrogen bonding and hydrophobic interactions, which forms a dense, low-permeability protein network that effectively impedes gas molecule diffusion [27].

Figure S6 presents the XRD patterns of PLA-Zein composite film, CL-AuNPs@CD-MOF, and CL-AuNPs@CD-MOF/PLA-Zein composite film. CL-AuNPs@CD-MOF exhibited characteristic CD-MOF peaks at 2θ = 4.0°, 5.7°, 6.9°, 13.4°, and 16.7°, along with metallic gold peaks at 38.0°, 44.2°, 64.5°, and 77.4°, corresponding to the (111), (200), (220), and (311) crystallographic planes, respectively. The PLA-Zein composite film displayed diffraction peaks at 15°, 16°, 19°, and 21°, with weaker peaks at 15° and 21° assigned to the (010) and (015) planes, and stronger peaks at 16° and 19° corresponding to the (200)/(110) and (203) planes of α-type crystals [74]. These characteristic peaks were retained in the CL-AuNPs@CD-MOF/PLA-Zein composite film, confirming that CL-AuNPs@CD-MOF incorporation did not disrupt the film’s crystallinity.

FTIR analysis (Figure S7) confirmed the characteristic absorption bands of each component. CL-AuNPs@CD-MOF displayed peaks at ~3500 cm^−1^ (O–H stretching), 2900 cm^−1^ (C–H stretching), 1750 cm^−1^ (carbonate ester linkages), and a complex fingerprint region (1000–1500 cm^−1^) attributed to C–O–C glycosidic bonds and C–OH groups. The PLA-Zein and composite films showed similar spectral profiles, including a broad O–H band at ~3500 cm^−1^, a carbonyl peak at 1755 cm^−1^, N–H/C–N vibrations at ~1540 cm^−1^, C–H stretching at 1458 and 1358 cm^−1^, C–O ester stretching at 1180 and 1084 cm^−1^ [25], and peaks at 870 and 754 cm^−1^ corresponding to amorphous and crystalline regions, respectively [75]. The absence of significant peak shifts, intensity changes, or new absorption bands in CL-AuNPs@CD-MOF/PLA-Zein composite film showed that CL-AuNPs@CD-MOF was physically incorporated without altering the molecular structure of the polymer matrix [75].

### 3.6. Mechanical Properties of CL-AuNPs@CD-MOF/PLA-Zein Composite Film

**Figure 7:**
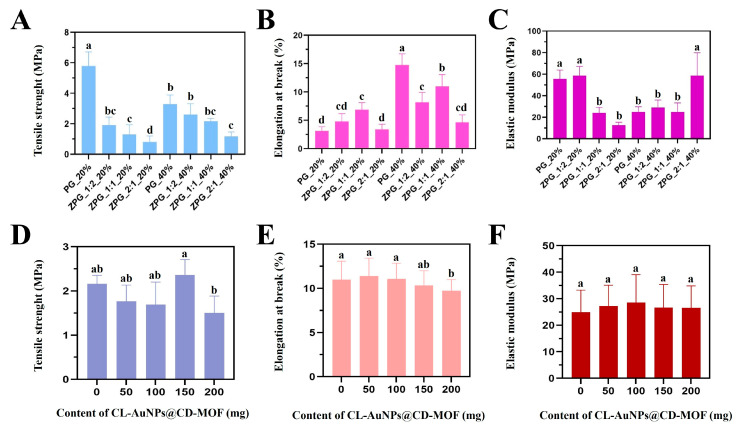
Tensile test of PLA-Zein composite film: (**A**) Tensile strength. (**B**) Elongation at break. (**C**) Elastic modulus. Tensile test of CL-AuNPs@CD-MOF/PLA-Zein composite film: (**D**) Tensile strength. (**E**) Elongation at break. (**F**) Elastic modulus. Values are expressed as mean ± SD. Different lowercase letters above bars indicate statistically significant differences among groups (one-way ANOVA with Tukey’s post-hoc test, *p* < 0.05); bars sharing the same letter are not significantly different.

Mechanical properties of PLA-Zein composite films are presented in Figure 7A–C. Tensile strength decreased with increasing zein content, dropping from 1.91 MPa at a 1:2 zein:PLA ratio to 0.8 MPa at 2:1, while higher PEG content improved tensile strength, rising from 1.30 to 2.16 MPa as PEG increased from 20% to 40% at a constant 1:1 zein:PLA ratio (Figure 7A) [76]. Elongation at break increased consistently with PEG content, from 6.88% to 10.98% at the 1:1 ratio, reflecting PEG’s plasticizing effect on polymer chain mobility (Figure 7B) [35,77]. Elastic modulus showed positive correlations with both zein and PEG contents, indicating their contribution to film stiffness (Figure 7C). Upon incorporation of CL-AuNPs@CD-MOF, tensile strength was unaffected at 50 and 100 mg loadings but increased notably at 150 mg, suggesting optimal matrix reinforcement at this concentration. Beyond 150 mg, tensile strength declined, likely due to MOF particle agglomeration creating stress concentration points that disrupt polymer matrix continuity (Figure 7D) [71,78]. Elongation at break and elastic modulus remained largely unchanged across all CL-AuNPs@CD-MOF concentrations (Figure 7E,F), indicating that the addition selectively influences tensile resistance without significantly affecting film flexibility or stiffness. Overall, based on the optimization results, the ZPG_1:1_40% formulation incorporating 150 mg (9.38 wt.%) of CL-AuNPs@CD-MOF powder was identified as the optimal composition and was therefore selected for subsequent barrier properties characterization and antibacterial application studies. Compared with the AgNPs@γ-CD-MOFs/PLA film reported by Liang et al. [26], which exhibited substantially higher tensile strength (10–16 MPa) owing to the inherent rigidity of the PLA-dominant matrix, the CL-AuNPs@CD-MOF/PLA-Zein film developed in this study showed lower tensile strength (0.8–2.16 MPa) but markedly superior elongation at break (6.88–10.98% vs. 2.5–3.8% for Liang et al.), attributable to the combined plasticizing effects of zein and PEG-4000 on polymer chain mobility. These results indicate that while the PLA-only matrix of Liang et al. confers greater tensile rigidity, the PLA-Zein co-matrix employed in the present study yields a more flexible and ductile film.

### 3.7. Barrier Properties of CL-AuNPs@CD-MOF/PLA-Zein Composite Film

**Figure 8:**
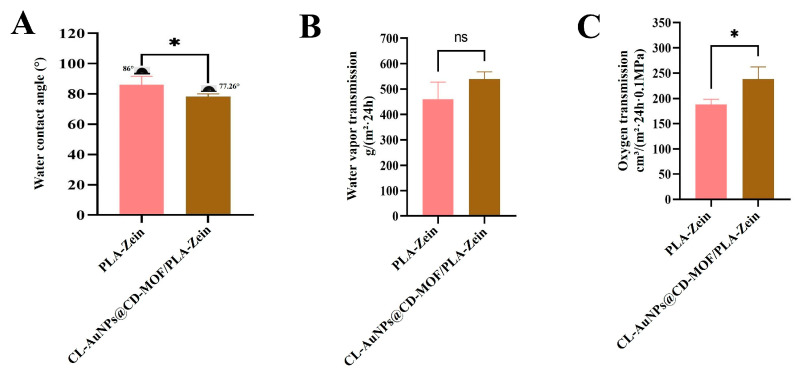
Barrier properties of PLA-Zein, and CL-AuNPs@CD-MOF/PLA-Zein composite film. (**A**) Water contact angle. (**B**) Water vapor transmission rate. (**C**) Oxygen transmission rate. Statistical significance between PLA-Zein and CL-AuNPs@CD-MOF/PLA-Zein groups is indicated as ns (not significant) (*p* ≥ 0.05) and * (*p* < 0.05).

Barrier properties of PLA-Zein and CL-AuNPs@CD-MOF/PLA-Zein composite films were evaluated (Figure 8). Incorporation of CL-AuNPs@CD-MOF reduced the water contact angle from 80° to 77.26° (Figure 7A), indicating increased hydrophilicity attributed to hydroxyl groups on the γ-CD-MOF surface [79]. Water vapor transmission rate increased from 460.36 to 539.44 g/(m^2^·24 h), consistent with this hydrophilicity trend (Figure 8B). Oxygen permeability also increased from 187.84 to 235.90 cm^3^/(m^2^·24 h·0.1 MPa), likely due to reduced crystallinity in the composite film (Figure 8C) [80]. Overall, the moderate hydrophilicity, controlled moisture transmission, and relatively high oxygen permeability of the composite film are advantageous for fresh produce packaging, preventing moisture condensation while supporting fruit and vegetable respiration during storage [81,82]. Compared to the PLA/PEG-4000 system reported by Liang et al. [26], which exhibited an oxygen transmission rate (OTR) of 1204 cm^3^/m^2^·d·0.1 MPa and a water vapor transmission rate (WVTR) of 1710.27 g/m^2^·24 h, the PLA-Zein matrix in the present study demonstrated substantially improved barrier performance despite the observed dispersed pahse in SEM. Studies have reported that incorporating zein as a coating or interlayer into packaging matrices substantially improves oxygen barrier performance. Hamdani et al. [83] demonstrated that a PVOH/zein bilayer coating on bleached kraft paper achieved an OTR as low as 128.0 ± 14.7 cc/m^2^·day, representing a 39-fold improvement over uncoated paper, while Liao et al. [84] reported that electrostatic deposition of a zein layer onto deacetylated chitin nanocrystal film reduced the OTR from 896.3 ± 35.9 to 769.4 ± 47.4 cm^3^/m^2^·d·bar, attributing this improvement to the formation of a more compact, pore-free surface structure. This enhancement is primarily attributed to zein’s compact α-helical secondary structure, which is stabilized by extensive intramolecular hydrogen bonding and hydrophobic interactions, forming a dense, low-permeability protein network that effectively impedes gas and water molecule diffusion [85].

### 3.8. Release Study of Gold from CL-AuNPs@CD-MOF/PLA-Zein Composite Film

**Figure 9:**
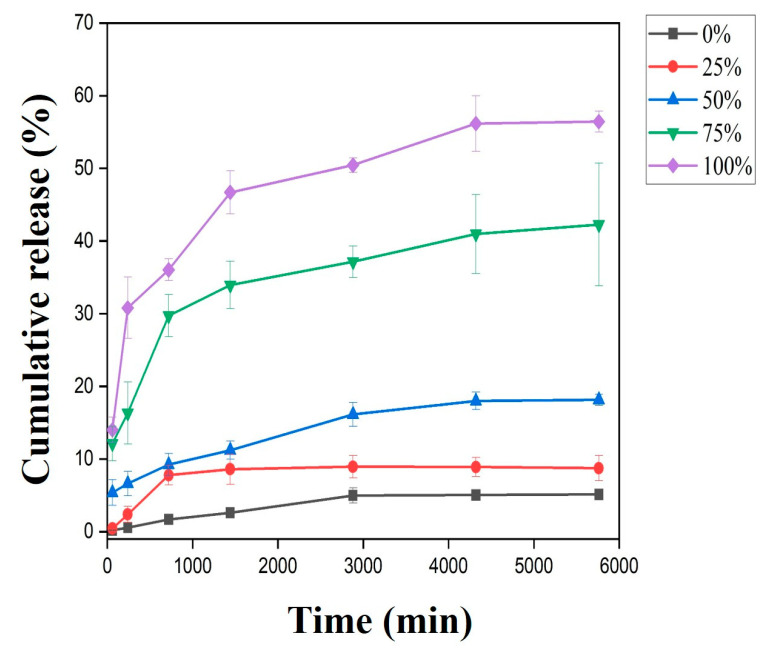
The release curve of gold ions from CL-AuNPs@CD-MOF/PLA-Zein composite film at different times and humid conditions.

The gold ion release profiles are presented in Figure 9. At equivalent time points, the cumulative release rate of the composite film increased proportionally with relative humidity. Under dry conditions (0% RH), minimal gold ion release was observed, reaching only 5.12% after 5760 min. When the humidity was elevated to 50% RH, the cumulative release increased to 18.14% at the same time point. Under saturated conditions (100% RH), the release rate was substantially higher, achieving 56.43% cumulative release at 5760 min. These results confirm that the CL-AuNPs@CD-MOF/PLA-Zein composite film exhibits a clear humidity-responsive release behavior [28]. This controlled release mechanism is attributed to the moisture-sensitive nature of the CL-AuNPs@CD-MOF framework embedded within the film matrix. As environmental humidity increases, water molecules progressively penetrate the polymeric matrix and interact with the CD-MOF scaffold, disrupting host–guest interactions and promoting the gradual diffusion of gold ions into the surrounding environment. This self-regulating behavior is particularly relevant for active food packaging applications, where humidity levels fluctuate dynamically during storage [86]. For instance, fresh produce such as mushrooms generates moisture through respiration, creating localized high-humidity microenvironments within sealed packaging. In this context, the composite film could autonomously respond to rising humidity by increasing antimicrobial agent release precisely when and where microbial proliferation risk is highest, effectively functioning as a self-triggered antimicrobial system. Furthermore, the graded release observed across 0%, 50%, and 100% RH suggests that the film can modulate its antimicrobial output in a proportional and sustained manner, avoiding the burst release limitations commonly associated with conventional active packaging systems. Collectively, these findings position the CL-AuNPs@CD-MOF/PLA-Zein film as a promising stimuli-responsive packaging material capable of delivering on-demand antimicrobial protection aligned with the actual preservation needs of the packaged product [87].

### 3.9. In Vitro Antibacterial Study of CL-AuNPs@CD-MOF/PLA-Zein Composite Film

**Figure 10:**
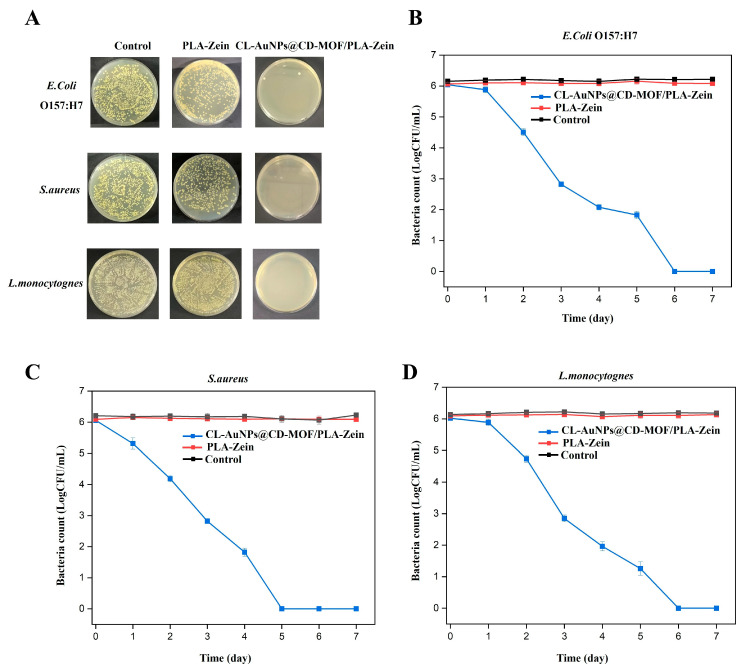
(**A**) Optical images of agar plates of PLA-Zein composite film, and CL-AuNPs@CD-MOF/PLA-Zein composite film (inoculated with 100 µL of 10^3^ dilution) against *E. coli* O157:H7, *S. aureus*, and *L. monocytogenes* after 7 days. Bacterial log reduction profiles of PLA-Zein composite film, and CL-AuNPs@CD-MOF/PLA-Zein composite film against: (**B**) *E. coli* O157:H7, (**C**) *S. aureus*, and (**D**) *L. monocytogenes*.

Figure 10 presents the time-dependent antibacterial activity of PLA-Zein and CL-AuNPs@CD-MOF/PLA-Zein composite films against *E. coli*, *S. aureus*, and *L. monocytogenes* over 7 days. The PLA-Zein film showed no antibacterial effect, with bacterial counts remaining stable at ~6.08, 6.09, and 6.13 log CFU/mL for *E. coli*, *S. aureus*, and *L. monocytogenes*, respectively, confirming the absence of inherent antimicrobial properties in the polymer matrix. In contrast, the CL-AuNPs@CD-MOF/PLA-Zein composite film exhibited progressive, broad-spectrum antibacterial activity against all tested strains. By day 3, reductions of 3.23, 3.21, and 3.18 log CFU/mL were recorded for *E. coli*, *S. aureus*, and *L. monocytogenes*, respectively. Complete bacterial elimination was achieved by day 5 for *S. aureus* and day 6 for both *E. coli* and *L. monocytogenes*, with *S. aureus* displaying slightly faster inactivation kinetics. This sustained efficacy is attributed to the controlled release of antimicrobial agents from the CL-AuNPs@CD-MOF component embedded in the polymer matrix. These results are consistent with previous studies reporting antimicrobial activity of bioactive-loaded PLA-Zein films, including eugenol-incorporated films producing inhibition zones of 3.33–6.83 mm against *S. aureus* and *E. coli* [35], and cinnamaldehyde-loaded PLA/Zein films yielding inhibition zones of 7 mm and 24 mm against *E. coli* and *S. aureus*, respectively [74]. Furthermore, Kalantari and Turner [88] reported that biosynthesized AuNPs demonstrated potent antimicrobial activity against both Gram-positive (*S. aureus*) and Gram-negative (*E. coli* and *P. aeruginosa*) bacterial strains, with MIC values ranging from 1.4 to 2.8 mg/mL. In addition, Guo et al. [28] demonstrated that ultra-small gold nanoparticles incorporated into a hydrophobic polydimethylsiloxane matrix (Au@CD-MOF/PDMS) achieved complete elimination of *E. coli* and *S. aureus* within 24 h of exposure.

### 3.10. Preservation Study of CL-AuNPs@CD-MOF/PLA-Zein Composite Film on Agaricus bisporus

**Figure 11:**
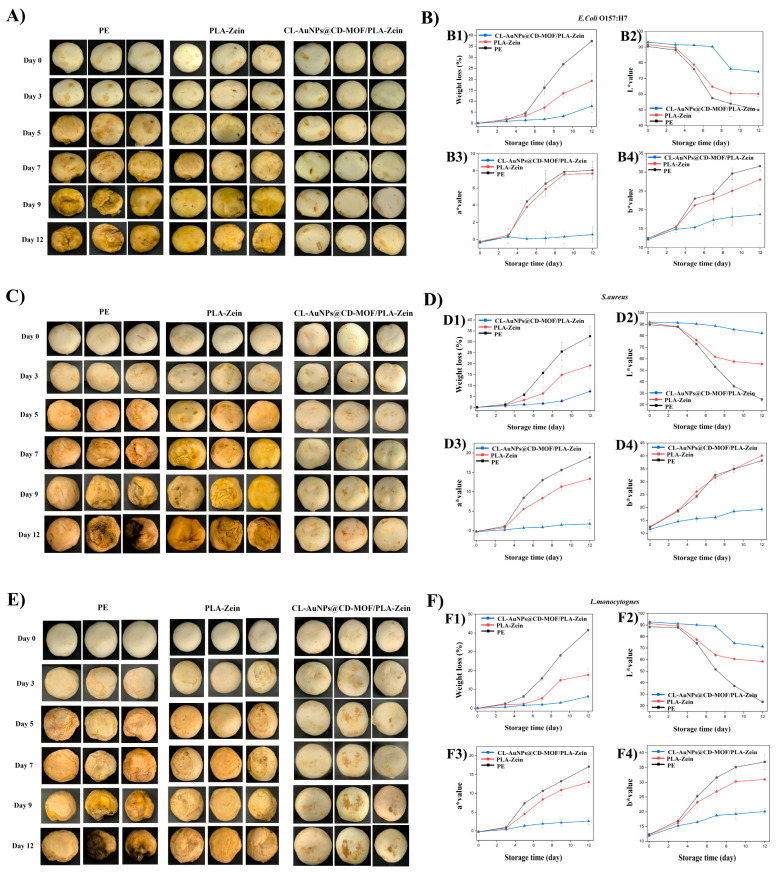
Storage performance of bacteria-inoculated *Agaricus bisporus* packaged with PE, PLA-Zein, and CL-AuNPs@CD-MOF/PLA-Zein composite films over 12 days. Visual appearance of *Agaricus bisporus* inoculated with (**A**) *E. coli* O157:H7. (**B**) Quality properties of *Agaricus bisporus* inoculated with *E. coli* O157:H7. (**C**) Visual appearance of *Agaricus bisporus* inoculated with *S. aureus*. (**D**) Quality properties of *Agaricus bisporus* inoculated with *S. aureus*. (**E**) Visual appearance of *Agaricus bisporus* inoculated with *L. monocytogenes*. (**F**) Quality properties of *Agaricus bisporus* inoculated with *L. monocytogenes*. L* (lightness, 0 = black to 100 = white), a* (green to red axis), and b* (blue to yellow axis) represent the surface color of *Agaricus bisporus* caps.

Figure 11 illustrates the quality attributes of mushrooms packaged with PE, PLA-Zein, and CL-AuNPs@CD-MOF/PLA-Zein composite films during storage. At day 0, all samples appeared white and firm. Over time, PE and PLA-Zein-protected mushrooms showed progressive color deterioration, with *E. coli*-inoculated samples developing browning and *S. aureus* and *L. monocytogenes*-inoculated samples exhibiting reddish discoloration. From day 7 onward, marked shrinkage and physical deterioration were observed in these groups. In contrast, CL-AuNPs@CD-MOF/PLA-Zein-protected mushrooms retained their appearance throughout storage, with only minimal surface spotting on *S. aureus* and *L. monocytogenes*-inoculated samples. Weight loss diverged significantly from day 5 onward. In the *E. coli* group, PE and PLA-Zein-protected mushrooms reached 37.32% and 19.60% weight loss by day 12, respectively, while CL-AuNPs@CD-MOF/PLA-Zein-protected samples lost only 7.72%. Color parameters followed similar trends across all contamination groups. In the *S. aureus* group, lightness (L*) at day 12 dropped to 24.64 and 55.51 for PE and PLA-Zein-protected mushrooms, respectively, while composite film-protected samples retained a value of 82.38. Redness (a*) and yellowness (b*) increased substantially in PE and PLA-Zein groups, while composite film samples maintained significantly lower values, indicating superior color stability. Complementary ΔE analysis (Figure S8) confirmed progressive color deterioration across all packaging groups, with PE films showing the greatest overall color deviation (ΔE: 45.73–72.56) and CL-AuNPs@CD-MOF/PLA-Zein films demonstrating the lowest values (ΔE: 12.45–22.94), indicating markedly superior color stability. These findings corroborate the L*, a*, and b* results and further support the contribution of the antimicrobial CL-AuNPs@CD-MOF component to the preservation of mushroom visual quality during refrigerated storage. While the superior color stability observed in mushrooms packaged with CL-AuNPs@CD-MOF/PLA-Zein films is consistent with the antimicrobial performance of the composite, it is acknowledged that oxidative enzymatic browning may also contribute to color deterioration independently of microbial activity; the absence of antioxidant activity assays in this study represents a limitation, and future work should incorporate such measurements to more comprehensively attribute the observed color preservation effects. The enhanced preservation performance of CL-AuNPs@CD-MOF/PLA-Zein is attributed to its antimicrobial activity, which progressively eliminates surface bacteria, and its balanced barrier properties that minimize moisture loss while maintaining adequate gas exchange for mushroom respiration [89]. In addition, Figure S9 describes the bacterial log reduction on *Agaricus bisporus* surface during storage time. Throughout the 12-day storage period, neither the PLA-Zein nor the conventional PE film demonstrated any antibacterial efficacy against *E. coli*, *S. aureus*, or *L. monocytogenes* on Agaricus bisporus, confirming the absence of intrinsic antimicrobial properties in these control films. In contrast, the CL-AuNPs@CD-MOF/PLA-Zein composite film exhibited sustained, time-dependent antibacterial activity against all three strains. By day 5, *S. aureus* showed the highest susceptibility (3.82 log CFU/mL reduction), followed by *L. monocytogenes* (2.41 log CFU/mL) and *E. coli* (2.21 log CFU/mL). By day 12, *S. aureus* was completely eliminated, while *E. coli* and *L. monocytogenes* were reduced to near-undetectable levels (<1 log CFU/mL), demonstrating the composite film’s robust antimicrobial efficacy throughout storage These findings are consistent with previous studies, including bacterial cellulose membranes with pomegranate peel extract that reduced microbial counts to 6.5 log CFU/g by day 15 [90]. Similarly, Zhang et al. [91] developed CEO-loaded mesoporous silica nanoparticle/potato starch (MSNP-CEO/PS) films via casting to protect post-*Agaricus bisporus* against *Mucor circinelloides* (CNRMA 03.0371) and *Mucor* sp. (FJ09). Liang et al. [26] demonstrated that AgNPs@γ-CD-MOF/PLA composite films effectively preserved mushroom appearance and color over 12 days against *Pseudomonas tolaasii*, a representative spoilage microorganism commonly associated with fresh mushrooms. While this represents a meaningful benchmark, the present study extends these findings by evaluating film performance against a broader spectrum of microorganisms, including foodborne pathogens (*E. coli* O157:H7, *S. aureus*, and *L. monocytogenes*) that pose significant contamination risks in fresh produce such as mushrooms. However, the microorganisms selected in our study (*E. coli*, *S. aureus*, and *L. monocytogenes*) represent relevant foodborne pathogens associated with mushroom contamination and public health risk; however, they do not fully reflect the typical spoilage microbiota of *Agaricus bisporus*, which includes fungi, yeasts, and *Pseudomonas* spp. The antimicrobial efficacy of the CL-AuNPs@CD-MOF/PLA-Zein composite film against these spoilage-associated microorganisms should be evaluated in future studies.

## 4. Conclusions

In this study, CL-AuNPs@CD-MOF was successfully synthesized and comprehensively characterized, confirming that the crosslinking process preserved the characteristic cubic morphology and crystalline structure of the CD-MOF framework. Crosslinking conditions were optimized to a CD-MOF:DPC weight ratio of 1:1, reaction time of 1080 min, and temperature of 80 °C, achieving both high material yield and controlled gold ion release. Importantly, crosslinking proved essential for transforming burst release behavior into sustained antimicrobial activity, enabling continuous inactivation of *E. coli* and *S. aureus* over 7 days, a performance unachievable with non-crosslinked AuNPs@CD-MOF. Incorporation of CL-AuNPs@CD-MOF into PLA-Zein films produced a composite packaging material with favorable mechanical and barrier properties. The moderate hydrophilicity, controlled water vapor transmission rate (539.44 g/m^2^·24 h), and oxygen permeability (235.90 cm^3^/m^2^·24 h·0.1 MPa) created an optimal microenvironment for fresh produce packaging, preventing moisture condensation while supporting aerobic respiration of the packaged produce. Antibacterial testing confirmed progressive elimination of *E. coli*, *S. aureus*, and *L. monocytogenes* over 7 days, while the control PLA-Zein film showed no antimicrobial activity, confirming that efficacy originated exclusively from the CL-AuNPs@CD-MOF component. Practical application on *Agaricus bisporus* inoculated with all three pathogens demonstrated effective bacterial reduction alongside superior preservation of mushroom quality, including significantly lower weight loss and better color stability (L*, a*, b*) compared to PE and PLA-Zein controls. These findings establish CL-AuNPs@CD-MOF/PLA-Zein as a promising multifunctional antimicrobial packaging platform with strong potential against spoilage and pathogenic microorganisms for practical food preservation applications. Despite these promising results, two key limitations of this study should be acknowledged: the absence of antioxidant activity assessment, which may lead to overinterpretation of the color preservation results, and the exclusive use of foodborne pathogens without inclusion of a representative mushroom spoilage organism such as *Pseudomonas tolaasii*, which limits direct comparability with ecologically relevant preservation conditions.

## Figures and Tables

**Table 1:** Kinetics model fitting.

Model	Regression Equation
Zero-order equation	Q = kt + k_0_
First-order kinetic equation	Q = k_0_(1 − e^−kt^)
Higuchi equation	Q = k × t^1/2^ + k_0_
Korsmeyer-Peppas equation	Q = (M_t_/M∞) = km t^n^

Note: Q is the cumulative release amount of gold ion at time t, k is the release rate constant, and k_0_ is the burst release constant.

**Table 2:** Kinetics model fitting results.

Model	Parameters	Values
Zero-order equation	R^2^	0.9666
	K	0.0059
	k_0_	5.3236
First-order kinetic equation	R^2^	0.9928
	K	0.0057
	k_0_	1.9905
Higuchi equation	R^2^	0.9772
	K	0.6588
	k_0_	5.375
Korsmeyer–Peppas equation	R^2^	0.9278
	K	0.5233
	k_0_	0.4049

## Data Availability

The original contributions presented in this study are included in the article and Supplementary Material. Further inquiries can be directed to the corresponding authors.

## Supplementary Materials

The following supporting information can be downloaded at https://www.mdpi.com/article/10.3390/foods15071164/s1, Table S1: Formulation matrix of PLA-Zein composite films prepared at varying zein-to-PLA mass ratios (1:2, 1:1, and 2:1) with PEG 400 as plasticizer at 20% and 40% (w/w, based on total polymer weight); Figure S1: (A) TEM of CL-AuNPs@CD-MOF particles dispersed in water; (B) particle size distribution; Figure S2: XPS spectra of CL-AuNPs@CD-MOF (A); k2p and C 1s, (B) Au4f; Figure S3: TGA patterns of γ-CD, CD-MOF, CL-AuNPs@CD-MOF; DTGA of CL-AuNPs@CD-MOF (A). DTGA comparison of γ-CD, CD-MOF, and CL-AuNPs@CD-MOF (B; Figure S4: (A) Optical images of agar plates (inoculated with 100 µL of 10^3^ dilution) of CL-AuNPs@CD-MOF and AuNPs@CD-MOF against *E. coli* O157:H7. (B) Optical images of agar plates of CL-AuNPs@CD-MOF and AuNPs@CD-MOF against *S.aureus*; Figure S5: Cytotoxicity assays of CL-AuNPs@CD-MOF tested on airway smooth muscle cells (ASMCs). No significant difference was observed among (P ≥ 0.05); Figure S6: PXRD patterns of PLA-Zein composite film, CL-AuNPs@CD-MOF, and CL-AuNPs@CD-MOF/PLA-Zein composite film; Figure S7: FTIR spectra of PLA-Zein composite film, CL-AuNPs@CD-MOF, and CL-AuNPs@CD-MOF/PLA-Zein composite film; Figure S8: Total color difference (ΔE) of *Agaricus bisporus* packaged with CL-AuNPs@CD-MOF/PLA-Zein, PLA-Zein, and PE films during 12 days of storage under (A) *E. coli* O157:H7; (B) *S. aureus*; (C) *L.monocytogenes* inoculation. Figure S9: Bacteria count measurement on *Agaricus bisporus* on 12 days of storage. (A) Optical images of agar plates of PE film, PLA-Zein composite film, and CL-AuNPs@CD-MOF/PLA-Zein composite film against *E. coli* O157:H7. (B) Bacterial log reduction profiles against *E. coli* O157:H7. (C) Optical images of agar plates of PE film, PLA-Zein composite film, and CL-AuNPs@CD-MOF/PLA-Zein composite film against *S.aureus*. (D) Bacterial log reduction profiles against *S.aureus*. (E) Optical images of agar plates of PE film, PLA-Zein composite film, and CL-AuNPs@CD-MOF/PLA-Zein composite film against L. monocytogenes. (F) Bacterial log reduction profiles against *L. monocytogenes*.
