# Supplementary material for: Crosslinked-AuNPs@CD-MOF Incorporated into PLA-Zein Composite Film with Humidity-Responsive Antimicrobial Release for Agaricus bisporus Preservation
Source: Foods. 2026 Mar 30;15(7):1164. doi: 10.3390/foods15071164 (PMC13073409; doi:10.3390/foods15071164)
Supplement: Supplementary file 1 [file foods-15-01164-s001.zip › foods-4196721-supplementary.pdf]

Supporting information

Crosslinked-AuNPs@CD-MOF Incorporated into PLA-ZeinComposite Film with Humidity-Responsive Antimicrobial Release for Agaricus bisporus Preservation

Tahirou Sogore [1], Meimei Guo [1], Jin Huang [1], Xinyu Liao [3], Tian Ding [1,3*] and Mofei Shen [2*]

1 College of Biosystems Engineering and Food Science, Zhejiang University, Hangzhou, 310058, China.

2 Department of Food Science and Engineering, Zhejiang University of Technology, Hangzhou, 310014, Zhejiang, People's Republic of China.

3 Future Food Laboratory, Innovation Center of Yangtze River Delta, Zhejiang University, Jiaxing, 314100, China.

* Correspondence: tding@zju.edu.cn, mfshen@zjut.edu.cn

Table S1. Formulation matrix of PLA-Zein composite films prepared at varying zein-to-PLA mass ratios (1:2, 1:1, and 2:1) with PEG 400 as plasticizer at 20% and 40% (w/w, based on total polymer weight).

Sample Code	Zein:PLA Ratio	PEG 400 Content (w/w)	Description
PG_20%	0:1 (Pure PLA)	20%	Pure PLA with 20% PEG
PG_40%	0:1 (Pure PLA)	40%	Pure PLA with 40% PEG
ZPG_1:2_20%	1:2	20%	Zein-PLA blend (1:2) with 20% PEG
ZPG_1:1_20%	1:1	20%	Zein-PLA blend (1:1) with 20% PEG
ZPG_2:1_20%	2:1	20%	Zein-PLA blend (2:1) with 20% PEG
ZPG_1:2_40%	1:2	40%	Zein-PLA blend (1:2) with 40% PEG
ZPG_1:1_40%	1:1	40%	Zein-PLA blend (1:1) with 40% PEG
ZPG_2:1_40%	2:1	40%	Zein-PLA blend (2:1) with 40% PEG

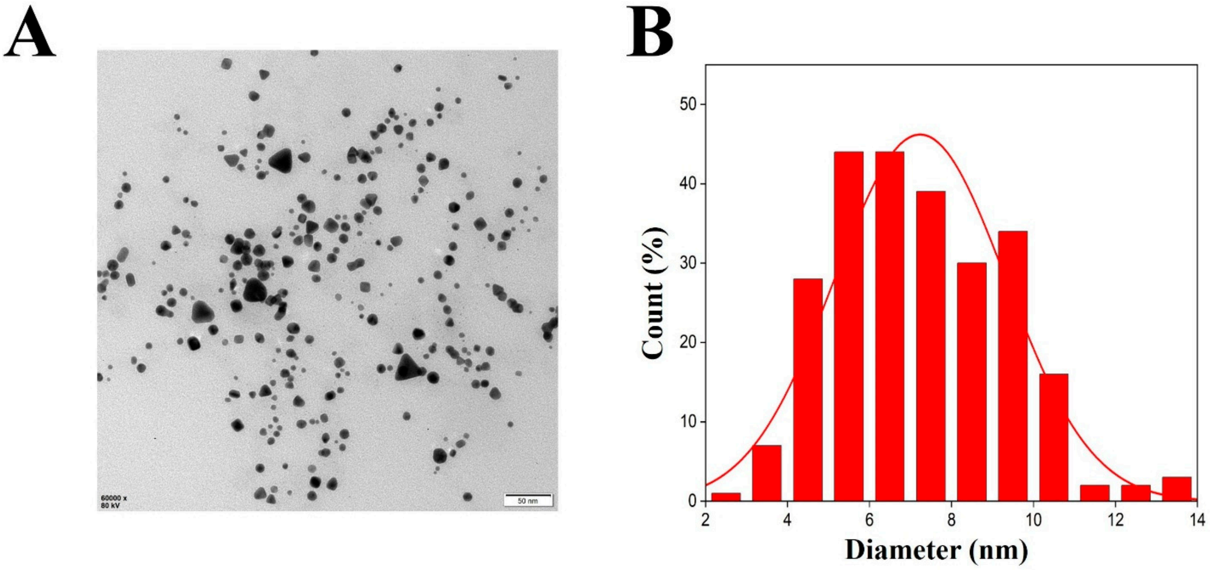

Figure S1. (A) TEM of CL-AuNPs@CD-MOF particles dispersed in water; (A) particle size distribution.

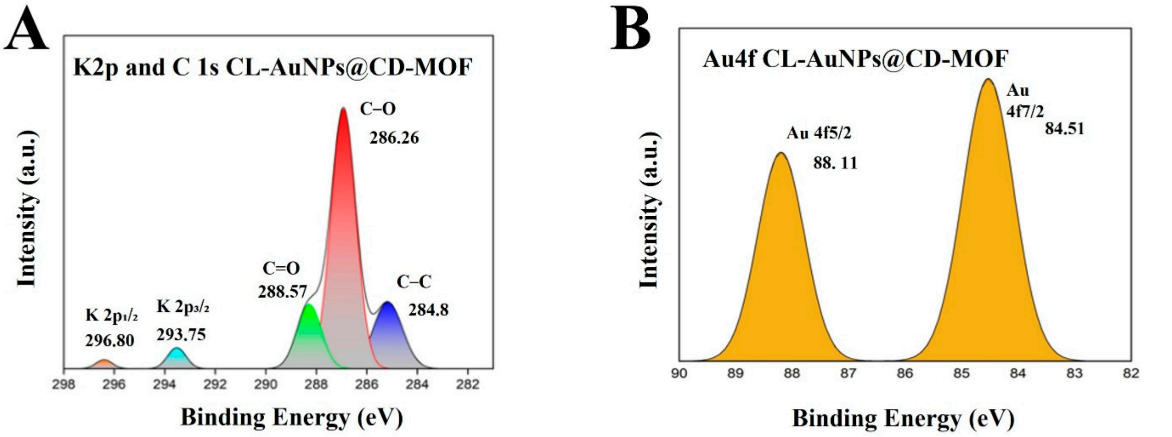

Figure S2. XPS spectra of CL-AuNPs@CD-MOF (A); k2p and C 1s, (B) Au4f.

The DTGA curves provided further insight into the thermal decomposition behavior of the materials (Figure S3). Pure γ-CD exhibited a single sharp decomposition peak at approximately 310°C, reflecting its rapid and well-defined thermal degradation characteristic of a crystalline structure. Upon formation of the CD-MOF, the decomposition profile broadened into two overlapping peaks at approximately 290°C and 350°C, indicating a more complex, stepwise

degradation attributed to the γ-CD/K^+ coordination framework. Notably, CL-AuNPs@CD-MOF displayed a near-flat DTGA curve across the entire temperature range, with no distinct decomposition peak, demonstrating a dramatically reduced rate of weight loss. This behavior confirms that crosslinking with DPC combined with AuNP incorporation significantly suppressed thermal decomposition, imparting superior thermal stability to the final material.

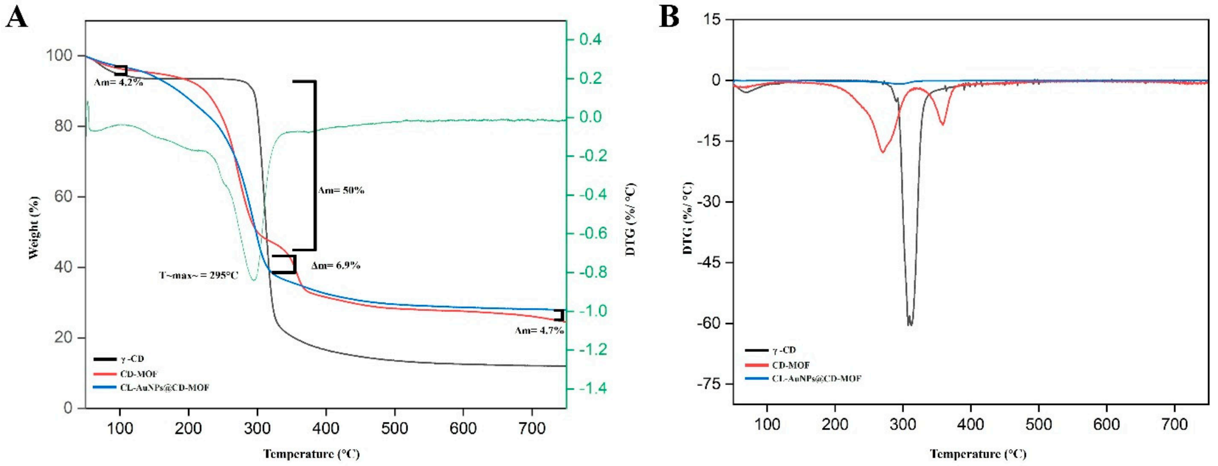

Figure S3. TGA patterns of γ-CD, CD-MOF, CL-AuNPs@CD-MOF; DTGA of CL-AuNPs@CD-MOF (A). DTGA comparison of γ-CD, CD-MOF, and CL-AuNPs@CD-MOF (B).

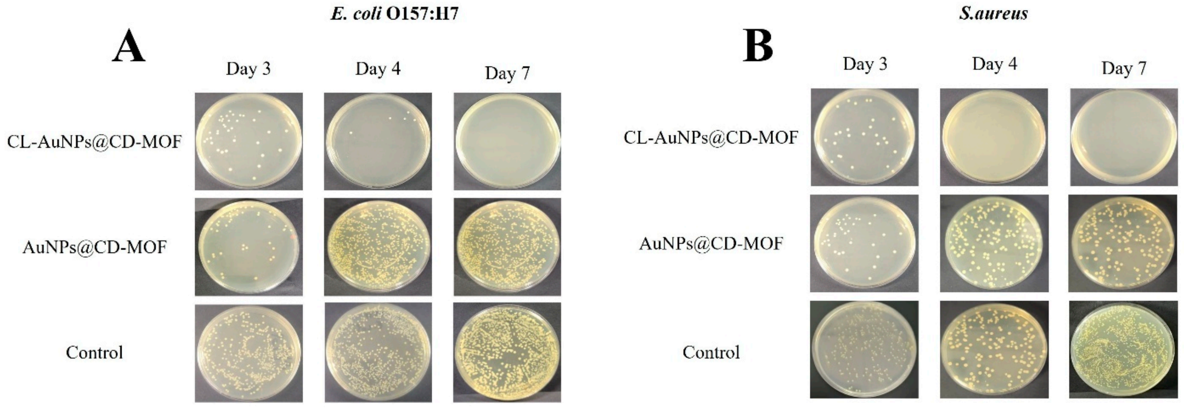

Figure S4. (A) Optical images of agar plates (inoculated with 100 µl of 10^3 dilution) of CL-AuNPs@CD-MOF and AuNPs@CD-MOF against *E. coli O157:H7*. (B) Optical images of agar plates of CL-AuNPs@CD-MOF and AuNPs@CD-MOF against *S.aureus*.

Cell viability of airway smooth muscle cells (ASMCs) exposed to CL-AuNPs@CD-MOF was evaluated to determine its cytotoxic profile, with findings illustrated in Figure S5. Viability exceeded 90% across all tested concentrations, confirming a favorable safety profile. Notably, the diphenyl carbonate-based crosslinking treatment applied to improve aqueous stability did not compromise the inherent biocompatibility of the CD-MOF scaffold, supporting the suitability of CL-AuNPs@CD-MOF for antimicrobial and biomedical applications [71].

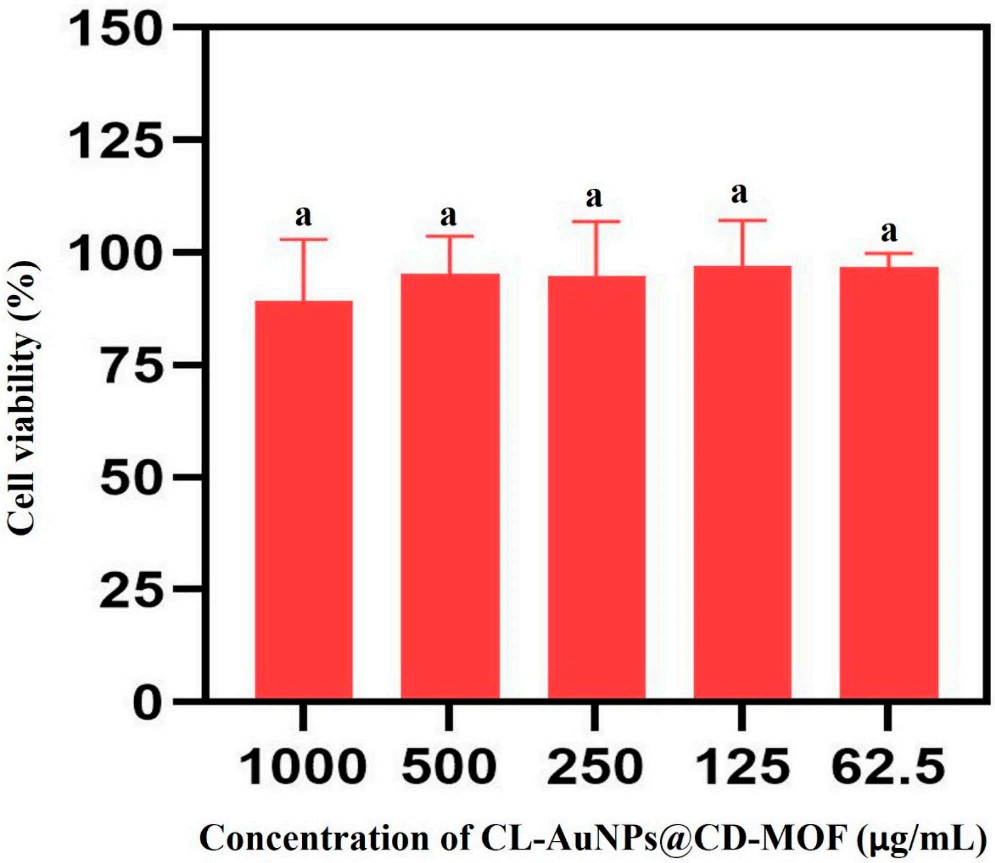

Figure S5. Cytotoxicity assays of CL-AuNPs@CD-MOF tested on airway smooth muscle cells (ASMCs). No significant difference was observed among ($P \geq 0.05$).

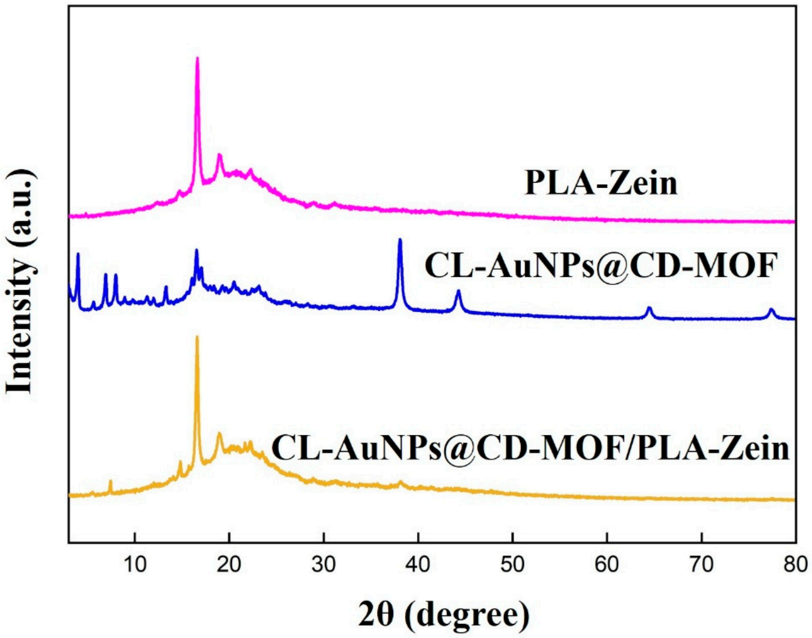

Figure S6. PXRD patterns of PLA-Zein composite film, CL-AuNPs@CD-MOF, and CL-AuNPs@CD-MOF/PLA-Zein composite film.

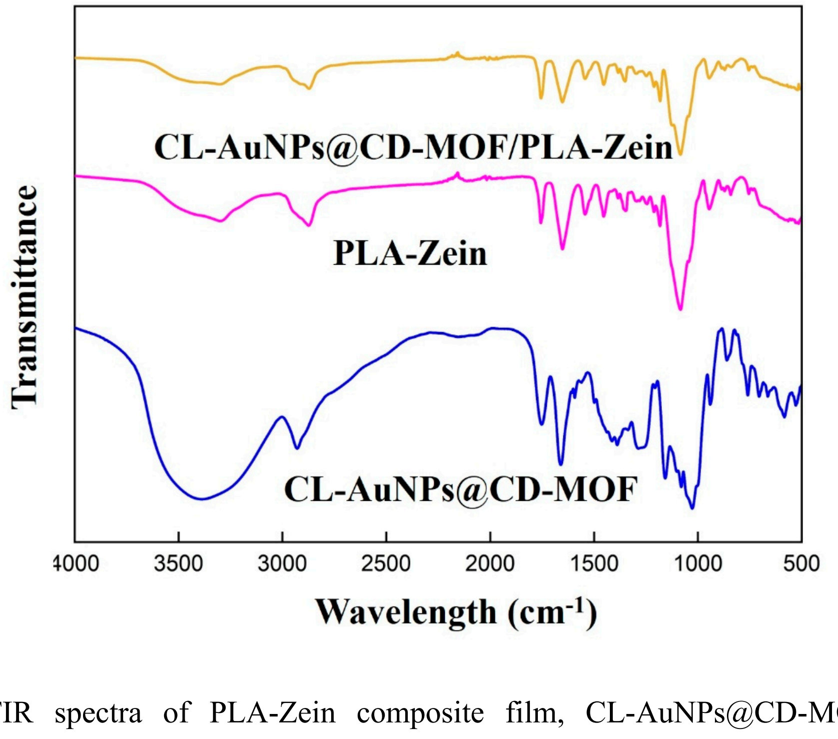

Figure S7. FTIR spectra of PLA-Zein composite film, CL-AuNPs@CD-MOF, and CL-AuNPs@CD-MOF/PLA-Zein composite film.

The total color difference (ΔE) of *Agaricus bisporus* mushrooms increased progressively over the 12-day storage period across all treatment groups, reflecting surface browning and color deterioration. Mushrooms packaged with PE film exhibited the most severe discoloration, reaching ΔE values of approximately 45.732 ± 4.07, 72.563 ± 2.76, and 71.75 ± 2.67 under *E. coli* O157:H7, *S. aureus*, and *L. monocytogenes* inoculation conditions, respectively. The PLA-Zein film showed intermediate performance, with final ΔE values of 36.20 ± 2.85, 47.13 ± 1.50, and 40.72 ± 0.84. In contrast, mushrooms packaged with CL-AuNPs@CD-MOF/PLA-Zein composite film maintained significantly lower ΔE values throughout storage (19.99 ± 2.65, 12.45 ± 0.75, and 22.94 ± 1.09, respectively), indicating markedly superior color retention. These results suggest that the incorporation of CL-AuNPs@CD-MOF into the PLA-Zein matrix effectively retarded discoloration, likely attributable to the antimicrobial properties of the AuNPs, thereby preserving the visual quality and commercial acceptability of the mushrooms.

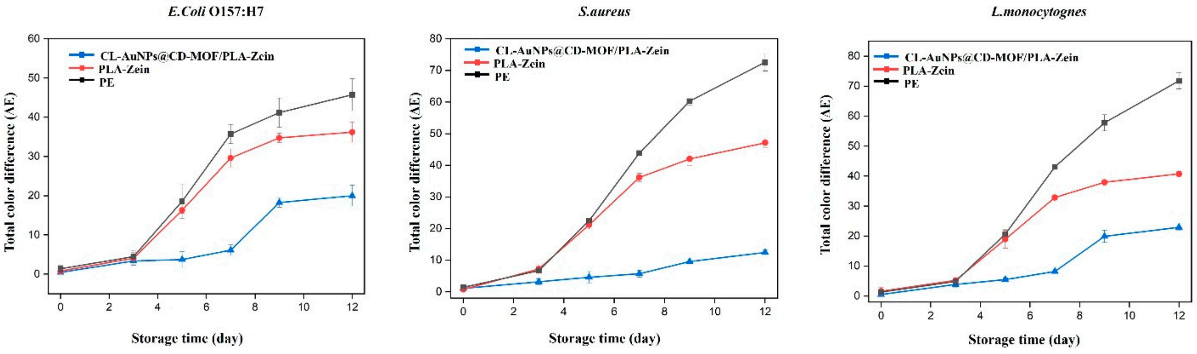

Figure S8. Total color difference (ΔE) of Agaricus bisporus packaged with CL-AuNPs@CD-MOF/PLA-Zein, PLA-Zein, and PE films during 12 days of storage under (A) *E. coli* O157:H7; (B) *S.aureus*; (C) *L.monocytogenes* inoculation.

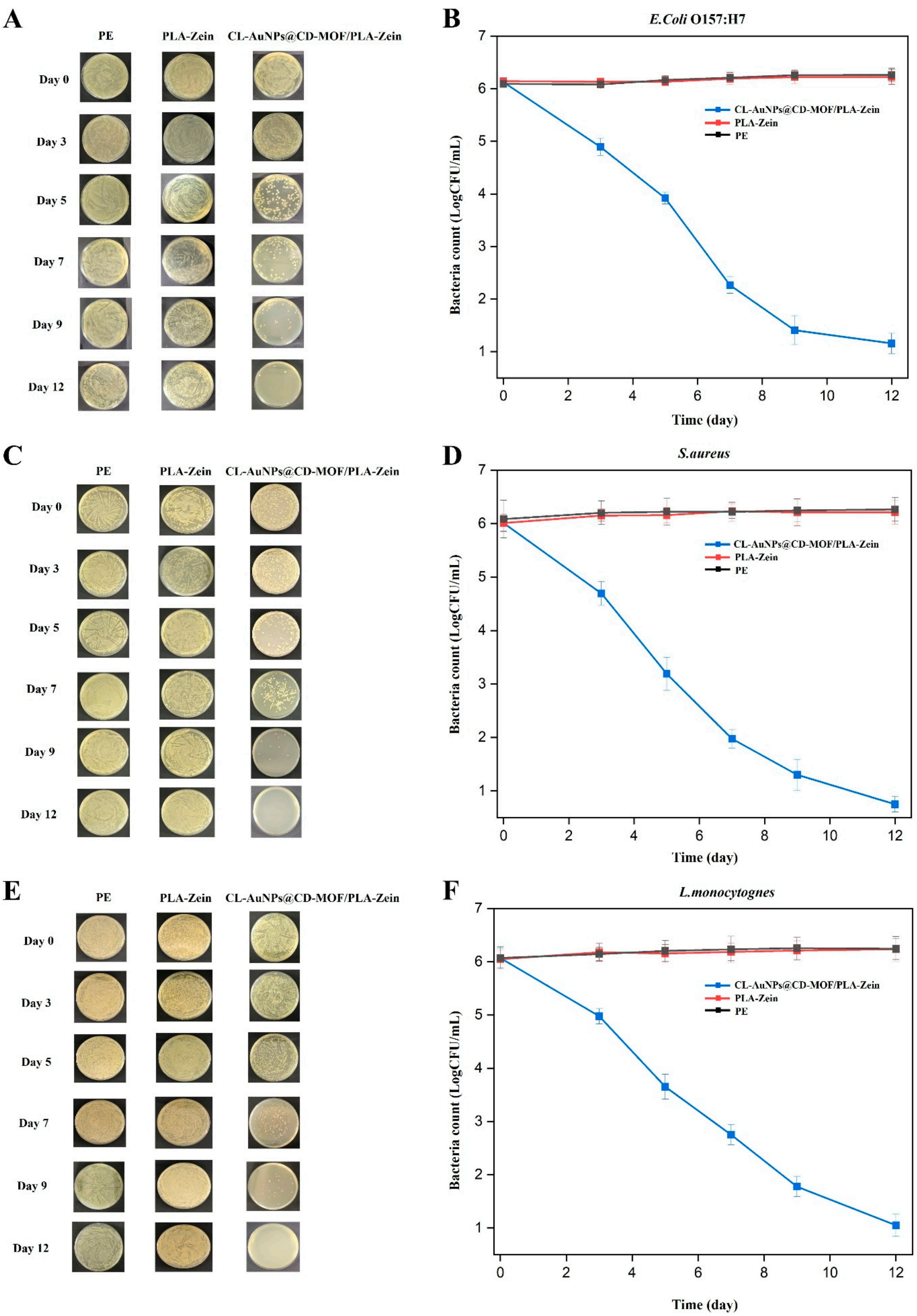
A
PE
PLA-Zein
CL-AuNPs@CD-MOF/PLA-Zein
Day 0
Day 3
Day 5
Day 7
Day 9
Day 12
B
E.Coli O157:H7
Bacteria count (LogCFU/mL)
Time (day)
CL-AuNPs@CD-MOF/PLA-Zein
PLA-Zein
PE
C
PE
PLA-Zein
CL-AuNPs@CD-MOF/PLA-Zein
Day 0
Day 3
Day 5
Day 7
Day 9
Day 12
D
S.aureus
Bacteria count (LogCFU/mL)
Time (day)
CL-AuNPs@CD-MOF/PLA-Zein
PLA-Zein
PE
E
PE
PLA-Zein
CL-AuNPs@CD-MOF/PLA-Zein
Day 0
Day 3
Day 5
Day 7
Day 9
Day 12
F
L.monocytognes
Bacteria count (LogCFU/mL)
Time (day)
CL-AuNPs@CD-MOF/PLA-Zein
PLA-Zein
PE

Figure S9. Bacteria count measurement on Agaricus bisporus on 12 days of storage. (A) Optical images of agar plates of PE film, PLA-Zein composite film, and CL-AuNPs@CD-MOF/PLA-Zein composite film against *E. coli O157:H7*. (B) Bacterial log reduction profiles against *E. coli* O157:H7. (C) Optical images of agar plates of PE film, PLA-Zein composite film, and CL-AuNPs@CD-MOF/PLA-Zein composite film against *S.aureus*. (D) Bacterial log reduction profiles against *S.aureus*. (E) Optical images of agar plates of PE film, PLA-Zein composite film, and CL-AuNPs@CD-MOF/PLA-Zein composite film against *L. monocytogenes*. (F) Bacterial log reduction profiles against *L. monocytogenes.*
